# Microcell-mediated chromosome transfer between non-identical human iPSCs

**DOI:** 10.1016/j.omtn.2024.102382

**Published:** 2024-11-05

**Authors:** Narumi Uno, Hitomaru Miyamoto, Kyotaro Yamazaki, Masaya Egawa, Hiroaki Kobayashi, Kanako Kazuki, Mitsuhiko Osaki, Teruhiko Suzuki, Shusei Hamamichi, Mitsuo Oshimura, Kazuma Tomizuka, Yasuhiro Kazuki

**Affiliations:** 1 Laboratory of Bioengineering, Faculty of Life Sciences, Tokyo University of Pharmacy and Life Sciences, 1432-1 Horinouchi, Hachioji, Tokyo 192-0392, Japan; 2 Department of Chromosome Biomedical Engineering, Integrated Medical Sciences, Graduate School of Medical Sciences, Tottori University, 86 Nishi-cho, Yonago, Tottori 683-8503, Japan; 3 Chromosome Engineering Research Group, Exploratory Research Center on Life and Living Systems (ExCELLS), National Institutes of Natural Sciences, 5-1 Higashiyama, Myodaiji, Okazaki, Aichi 444-8787, Japan; 4 Homeostatic Regulation, National Institute for Physiological Sciences, National Institutes of Natural Sciences (NINS), 5-1 Higashiyama, Myodaiji, Okazaki 444-8787, Japan; 5 Chromosome Engineering Research Center, Tottori University, 86 Nishi-cho, Yonago, Tottori 683-8503, Japan; 6 Division of Experimental Pathology, Faculty of Medicine, Tottori University, 86 Nishi-cho, Yonago, Tottori 683-8503, Japan; 7 Stem Cell Project, Tokyo Metropolitan Institute of Medical Science, Kamikitazawa, Setagaya-ku, Tokyo 156-8506, Japan

**Keywords:** MT: Delivery Strategies, paclitaxel, reversine, microcell-mediated chromosome transfer, induced pluripotent stem cell, disease model, human artificial chromosome, mouse artificial chromosome, trisomy, Klinefelter’s syndrome, triple X syndrome

## Abstract

Microcell-mediated chromosome transfer (MMCT) is anticipated as a unique strategy to manipulate numbers of chromosomes, including the generation of hyperaneuploidy syndrome models with human induced pluripotent stem cells (hiPSCs). Mouse A9/Chinese hamster ovary (CHO) cell libraries of human monochromosomal hybrids as chromosome donor cells frequently exhibit chromosomal rearrangement in the components. The generation of a new A9/CHO library is time-consuming and laborious. Here, we developed an MMCT method using hiPSCs as chromosome donor and recipient cells, through micronucleation using paclitaxel and reversine. Membrane fusion during the MMCT was mediated through interactions between the ecotropic viral envelope transiently expressed in chromosome donor cells and mCAT-1 in chromosome recipient cells. This approach involved tagging Chr21 and ChrY by CRISPR-Cas9 and transferring human/mouse artificial chromosomes, Chr21, ChrX, and ChrY, wherein there are no previous reports demonstrating a full-length introduction. Thus, a strategy that combing CRISPR-Cas9-mediated chromosome tagging and MMCT from hiPSCs as chromosome donor cells to hiPSCs as recipient cells systematically produced isogenic disease model hiPSCs with hyperaneuploidy. This approach allows the study of rare diseases and promises to provide new insights into early developmental mechanisms by introducing a comprehensive set of influential chromosomes/regions into hiPSCs.

## Graphical abstract

**Figure:**
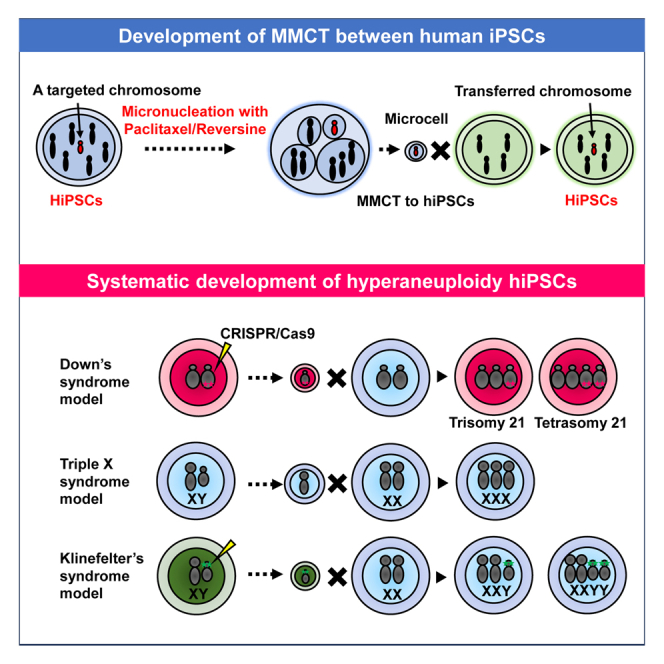

## Teaser

Kazuki and colleagues introduce an advanced MMCT method using hiPSCs as both chromosome donors and recipients, employing paclitaxel and reversine for efficient micronucleation. This refined method, coupled with CRISPR-Cas9 for chromosome tagging, allows for precise modeling of hyperaneuploidy, advancing our understanding of complex genetic diseases and developmental biology.

## Introduction

As a systematic approach to elucidating human chromosome-related biological phenomena, mouse A9/Chinese hamster ovary (CHO) cell libraries of human monochromosomal hybrids^1^^,^^2^^,^^3^ and A9/human cell hybrids^4^ have been used as chromosome donor cells (CDCs) as resources for microcell-mediated chromosome transfer (MMCT). The A9/CHO cell libraries consist of A9/CHO cells, each containing one of the human chromosomes 1 to 22 or X (ChrX), which have been tagged with drug-resistant genes using a random integration method in the originating human fibroblast cells.^3^ However, this system excludes chromosome Y (ChrY). Nevertheless, these human chromosomes can be transferred into cancer cell lines and human pluripotent stem cells (hPSCs) from A9/CHO cells through MMCT. Using these libraries, tumor suppressor gene mapping has been conducted by compensating for lost chromosomes or chromosome regions in cancer cells.^5^ By transferring chromosomes 8, 13, 18,^6^ and 21^7^ into hPSCs, isogenic trisomy syndrome model hPSCs have been established and used to elucidate disease mechanisms.^8^ Furthermore, an A9/human cell hybrid allowed the transfer of chromosomes that retained chromosomal repeats derived from patients with fragile X syndrome,^9^ a genetic disorder linked to ChrX, into target cells. Although A9/CHO cell libraries of human monochromosomal hybrids have been used, the chromosomes retained in these libraries frequently exhibit chromosome abnormalities.^10^ Furthermore, transferring intact human chromosomes is complex; the chromosomes of interest must be tagged with drug-resistant genes, fused with A9/CHO cells, and then introduced to target cells, particularly human induced pluripotent stem cells (hiPSCs), through MMCT.^11^ However, a previous study reported low MMCT efficiency in CHO/human cell hybrids, which involved first transferring chromosomes into CHO cells from the CHO/human hybrid cells, and subsequently retransferring them into hiPSCs, leading to the acquisition of hiPSCs with transferred chromosomes.^12^ In addition, the human fibroblast cells supplying the originating chromosomes have limited proliferative capacity, making the tagging of any chromosome with drug markers a highly challenging task, even when using CRISPR-Cas9.^13^

We thus focused on hiPSCs, which exhibit infinite proliferative capacity, maintain normal human chromosomes, and can be generated by reprogramming somatic cells.^14^ Moreover, universal reprogramming methods have been established, enabling the creation of disease model iPSCs from patient-derived fibroblasts,^15^^,^^16^^,^^17^ which are readily available from cell banks. Developing a novel MMCT method to transfer any chromosome from hiPSCs as CDCs directly to other hiPSCs as chromosome recipient cells (CRCs) allows the more straightforward generation of hyperaneuploidy disease models, genetic chromosomes disorder models, and cells containing familial chromosomes.

Here, we demonstrate an MMCT method that enables the transfer of chromosomes of various sizes/types, including human/mouse artificial chromosomes (HACs/MACs) (∼5 Mb),^18^ Chr21 (∼45 Mb), ChrX (∼154 Mb), and ChrY (∼60 Mb) (NIH-Human genome assembly GRCh38. p13) from hiPSCs to target cells, including other hiPSCs, using a recently developed method of inducing micronucleation with paclitaxel (PTX) and reversine (Rev).^19^^,^^20^ This MMCT approach enabled the induction of micronucleation in normal hiPSCs, which was traditionally challenging, and facilitated the introduction of chromosomes through MMCT; thus, normal hiPSCs can be used as CDCs. While direct MMCT from hiPSCs to CHO cells has been successful, the feasibility of direct MMCT from hiPSCs to human cells including hiPSCs, without A9/CHO cell hybrids had not been evaluated. Thus, we then determined the optimal drug treatment conditions that induce higher numbers of micronuclei and efficiency of MMCT. Chr21 and ChrY, which are representative examples of native chromosomes, were tagged with drug-resistant genes by CRISPR-Cas9 in hiPSCs. Moreover, we attempted the generation of isogenic hyperaneuploidy disease model hiPSCs for diseases such as Down syndrome (trisomy 21), triple X syndrome (47,XX,+X) and Klinefelter’s syndrome (47,XX,+Y). Here, we describe a method to use hiPSCs as a tool for systematic chromosome transfer to human cells. Using the conventional MMCT method, it took 6 months to acquire iPSCs that retained the target human chromosomes. In contrast, when using the MMCT method from hiPSCs to hiPSCs, we obtained hiPSCs with the introduced target chromosomes in just 2 months. This advancement significantly reduced the time required to generate iPSCs with specific chromosomal alterations, thus facilitating a faster and more efficient development of disease models.

## Results

### Optimization of treatment concentrations of PTX and Rev for MMCT from hiPSCs to human cell line HT1080

**Figure 1:**
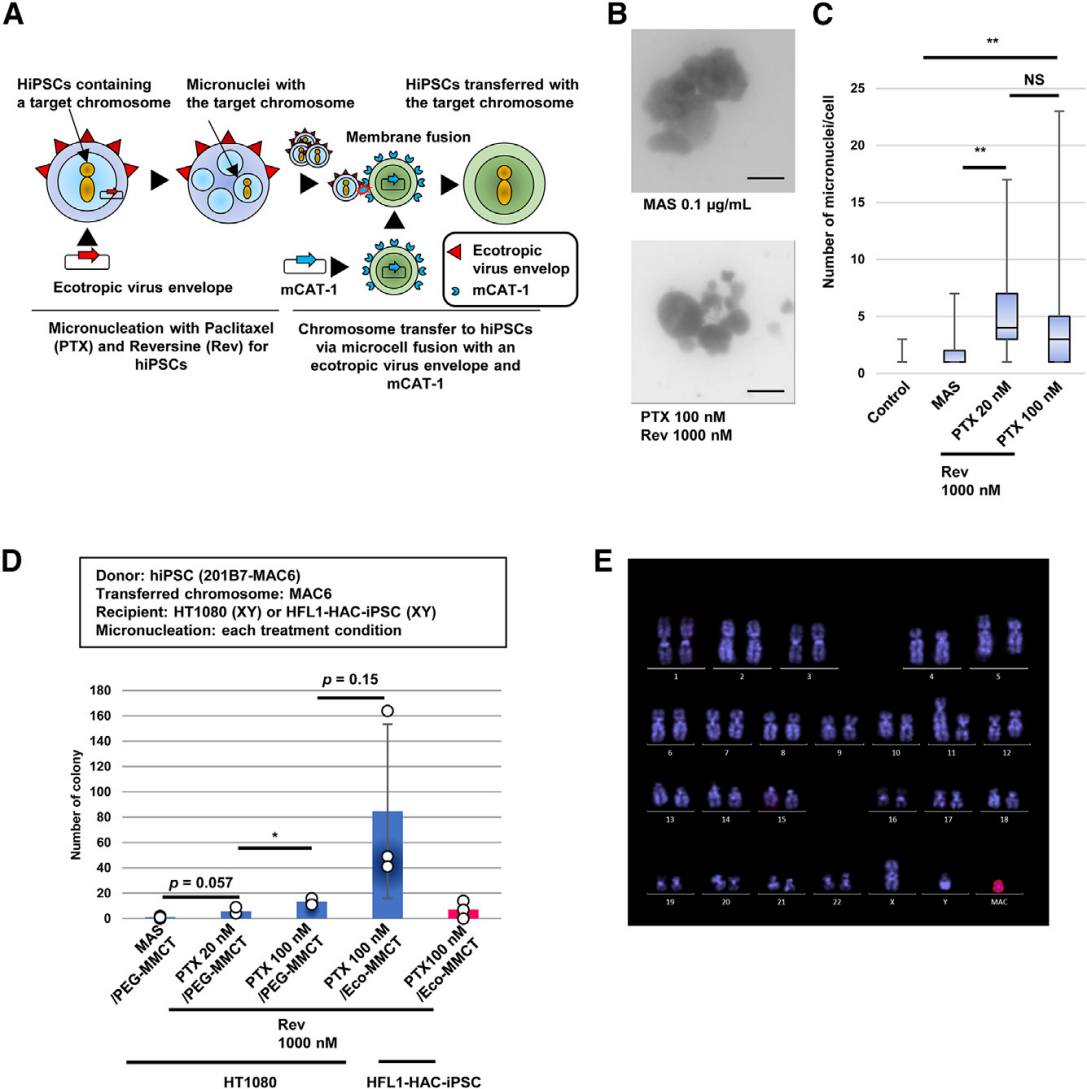
Optimization of treatment concentrations of paclitaxel and reversine for MMCT from hiPSC (A) Schematic illustration of micronucleation in hiPSCs with paclitaxel (PTX) and reversine (Rev) and application of Eco-MMCT using an ecotropic virus envelope and its receptor mCAT-1. (B) Giemsa-stained micronucler spreads of treated hiPSCs (201B7). Scale bars, 10 μm. (C) Number of micronuclei per cell under each treatment of hiPSCs. Line within the box marks the median. Box extends from the 25th percentile (Q1) to 75th percentile (Q3), representing the interquartile range (IQR). Whiskers extend from minimum to maximum values, *n* = 100 cells per group (∗∗*p* < 0.01, ANOVA). (D) Comparison of MMCT efficiency of MAC6 from hiPSCs (201B7-MAC6) to HT1080 or HFL1-HAC-iPSCs under various conditions. Blue bar indicates PEG-MMCT; red bar indicates Eco-MMCT (∗*p* < 0.05; Student’s t-test). Data are the mean ± SD (*n* = 3). The chromosome donor cells (CDCs), chromosome recipient cells (CRCs), and the transferred chromosomes are summarized in the frame at the top of the graph. (E) FISH analysis and karyotype of an HT1080 clone, transferred with MAC6. Fluorescence *in situ* hybridization (FISH) analysis of HT1080 transferred with MAC6. Blue indicates 4′,6-diamidino-2-phenylindole (DAPI) and red indicates mouse Cot-1 (MAC6).

MMCT involves inducing micronucleation of hiPSCs as CDCs and fusing microcells with CRCs (Figure 1A). We focused on PTX,^21^ a spindle stabilizer, and Rev,^22^ a spindle checkpoint inhibitor, for inducing micronuclei in hiPSCs. Metaphase arresting solution (MAS) was used to induce metaphase arrest for karyotyping of hiPSCs^23^ and the micronucleation ability was compared with PTX and Rev. Based on the previous study of MMCT from hiPSCs to CHO cells, we determined the treatment condition inducing the greater number of micronuclei for hiPSC line 201B7.^14^ We observed that MAS 0.1 μg/mL was insufficient to induce efficient micronucleation for 201B7,^14^ whereas PTX at 20 or 100 nM and Rev at 1,000 nM enabled the induction of a higher number of micronuclei in a cell (∗*p* < 0.01) (Figures 1B and 1C). We evaluated MMCT efficiency with 201B7 carrying MAC6^23^ (201B7-MAC6) containing a neomycin-resistant gene, as CDCs, and human fibrosarcoma cells (HT1080), as model CRCs, with polyethylene glycol (PEG) as a fusogen (PEG-MMCT).^24^ PTX 20 nM and Rev 1,000 nM showed an increase of the MMCT efficiency compared with MAS (*p* = 0.057), and PTX 100 nM and Rev 1,000 nM had a higher MMCT efficiency compared with PTX 20 nM and Rev 1,000 nM (∗*p* < 0.05) (Figure 1D; Table S1). These results suggested that the optimal concentrations for micronucleation and MMCT from hiPSCs to HT1080 were PTX 100 nM and Rev 1,000 nM.

In general, the MMCT efficiency is affected by the cell type of CRCs, and the efficiency in hiPSCs is lower than the efficiency in HT1080. To address this, the MMCT efficiency was enhanced using ecotropic (Eco)-MMCT compared with PEG-MMCT, as reported previously^25^ (Figure 1A). The co-culture of 201B7-MAC6 as CDCs expressing an ecotropic viral envelope and HT1080 as CRCs expressing its receptor, mCAT-1, resulted in membrane fusion inducing syncytium harboring multi-nuclei (Figures 1A and S1A). Consequently, Eco-MMCT showed a trend of higher efficiency compared to PEG-MMCT (*p* = 0.15) (Figure 1D; Table S1). Fluorescence *in situ* hybridization (FISH) analysis of the obtained HT1080-MAC6 clones with Eco-MMCT is summarized in Table S2. Each clone maintained MAC6 independent from host chromosomes. When hiPSCs were used as CRCs, plasmids expressing mCAT-1 were electroporated. Flow cytometry (FCM) analysis showed that >97% mCAT-1 positive population was obtained from the introduced hiPSCs (201B7, A6, and HFL1 SeV2-1) (Figure S1B). We successfully achieved the transfer of MAC6 from 201B7-MAC6 into HFL1-HAC-iPSCs,^26^ whereas the MMCT efficiency of transfer to HFL1-HAC-iPSCs was lower compared with HT1080 (XY) (Figure 1D; Table S1). The results of this efficiency evaluation suggest that Eco-MMCT is the optimal method for MMCT between hiPSCs.

### MMCT of ChrX as a representative native chromosome from hiPSCs to HT1080

**Figure 2:**
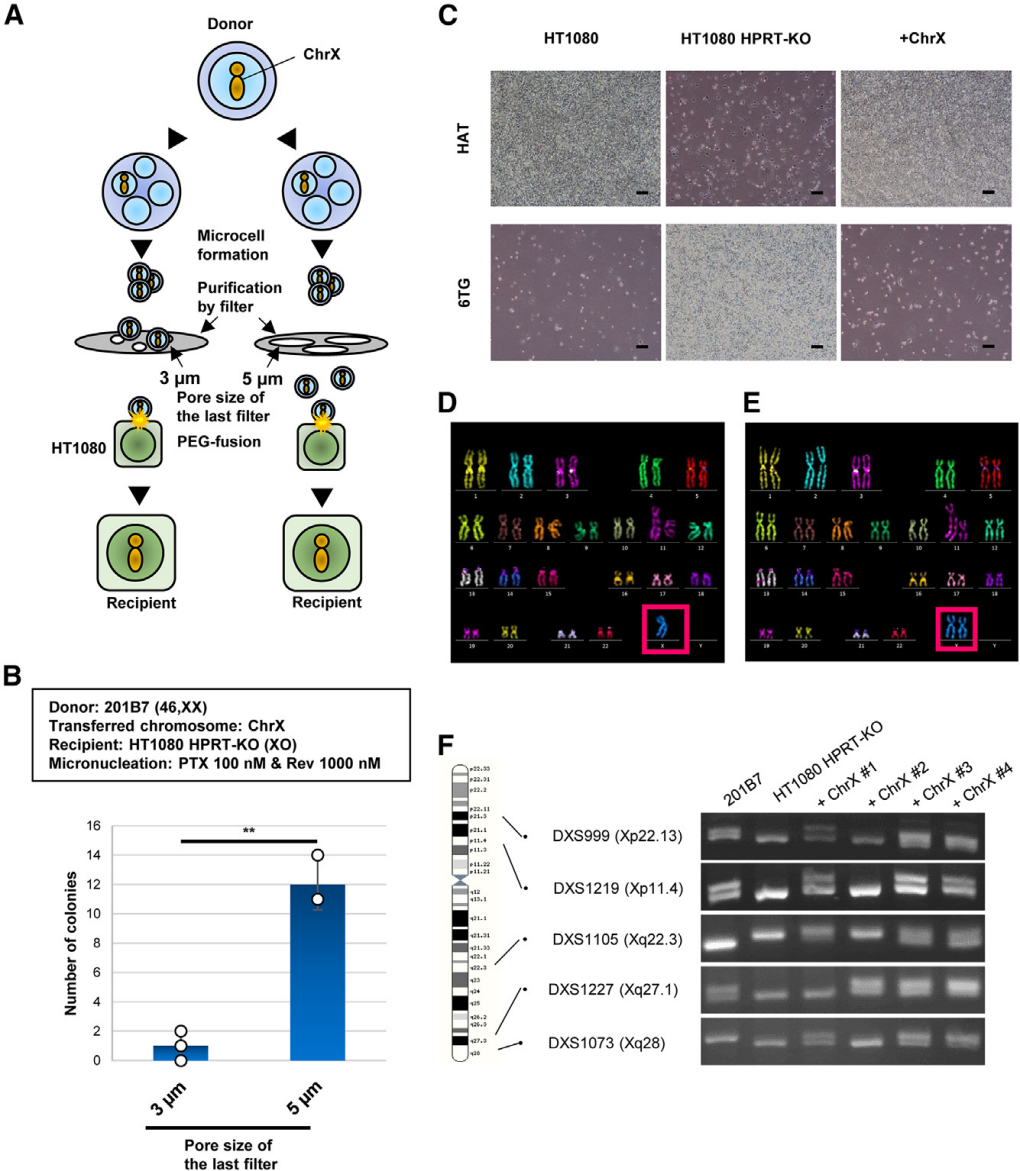
MMCT of endogenous ChrX from iPSCs to HT1080 (A) Schematic illustration of the relationship between filter pore size and microcell size and evaluation of the effect of filter pore size on microcell purification. Microcells containing ChrX might be larger than the 3-μm filter pore size and thus be lost during micronucleus purification. (B) Comparison of the minimum filter pore diameter used during microcell purification and the number of colonies obtained after MMCT. Data are the mean ± SD (*n* = 3). (C) Representative images of the drug resistance analysis of the *hypoxanthine-guanine phosphoribosyltransferase* (*HPRT*) gene on ChrX. The *HPRT* gene confers hypoxanthine-aminopterin-thymidine (HAT) resistance to cells and metabolizes 6-thioguanine (6TG) to produce cytotoxic metabolites, making it sensitive to 6TG. Thus, the HT1080 HPRT-KO cells transferred with ChrX recovered HAT resistance and 6TG sensitivity similar to wild-type HT1080 cells. Scale bars, 100 μm. (D and E) Multi-color FISH analysis of HT1080 HPRT-KO (XO) and a clone transferred with ChrX. Red frames indicate ChrX. (F) PCR with STS marker primers. STS markers showing polymorphisms on ChrX were selected for different band sizes between 201B7 and HT1080, and HT1080 clones acquired after ChrX transfer by MMCT were analyzed. The location of each STS marker is shown with the ideogram of ChrX. The results indicated that the clones +ChrX#3 and #4 might have an intact ChrX, but not +ChrX#2, which showed the loss of DXS1073. For Clone#1, DXS1227 might be defective, but it is not clear which of the STS marker polymorphisms, 201B7 or XX, was introduced.

We attempted to transfer a model endogenous chromosome, ChrX (∼154 Mb, larger than MAC6, ∼5 Mb), from hiPSCs to HT1080 using MMCT. MMCT includes the purification of microcells using a membrane filter to remove whole CDCs and larger microcells. Membrane filters with the pore diameters of 8 and 5 μm, or 8, 5, and 3 μm were used in sequence for purification, starting with the largest pore diameter. Recently, Mammel et al. reported the size of micronuclei/microcells correlated with the amount of DNA contained within, i.e., the size of the chromosome from which the micronuclei/microcell was derived.^27^ We hypothesized that microcells containing ChrX were less likely to pass through 3-μm pore diameter filters. We anticipated that only 8- and 5-μm pore diameter filters would improve the MMCT efficiency of ChrX (Figure 2A). Furthermore, ChrX contained an endogenous *hypoxanthine-guanine phospho-ribosyl-transferase* (*HPRT*) gene, which was used as a drug-resistant gene for hypoxanthine-aminopterin-thymidine (HAT) medium and has sensitivity to 6-thioguanine (6TG). ChrX was transferred from hiPSCs (201B7) to HT1080 HPRT-knockout (HPRT-KO) (XO)^23^ using PEG-MMCT with PTX 100 and Rev 1,000 nM. A total of three (average of 1 ± 1, SD) HT1080 clones with drug resistance were obtained when microcell suspensions were filtered through 8-, 5-, and 3-μm pore diameter filters (Figure 2B; Table S1). When only 8- and 5-μm pore diameter filters were used, a total of 36 drug-resistant colonies (average of 12 ± 1.73, SD) were obtained, demonstrating a significant improvement in chromosome introduction efficiency (∗*p* < 0.01) (Figure 2B; Table S1). From these findings, we set the minimum filtration membrane pore size to 5-μm pore diameter for the MMCT of ChrX. The obtained HT1080 HPRT-KO+ChrX was HAT resistant and underwent cell death due to the sensitivity of the *HPRT* gene to 6TG (Figure 2C). This indicated that an exogenous functional *HPRT* gene had been transferred. The results of multi-color FISH (mFISH) analysis also showed that the XO type of HT1080 HPRT-KO (Figure 2D) was converted to the XX type (Figure 2E). Furthermore, sequence-tagged site (STS) marker analysis was conducted to determine whether the conversion of HT1080 HPRT-KO from XO to XX resulted from uniparental disomy via ChrX duplication^15^ in the CRC or from the introduction of an intact ChrX (Figure 2F). ChrX#3 and #4 showed PCR amplification patterns similar to those of 201B7 across five STS markers suggesting the introduction of ChrX. In contrast, ChrX#1 and #2 displayed partial deletions, specifically, at DXS1105 (Xq22.3) and/or DXS1073 (Xq28) in the exogenous ChrX (Figure 2F). Thus, endogenous ChrX was transferrable from hiPSCs to HT1080.

### Chromosome tagging by CRISPR-Cas9 and MMCT of the targeted native Chr21 and ChrY

**Figure 3:**
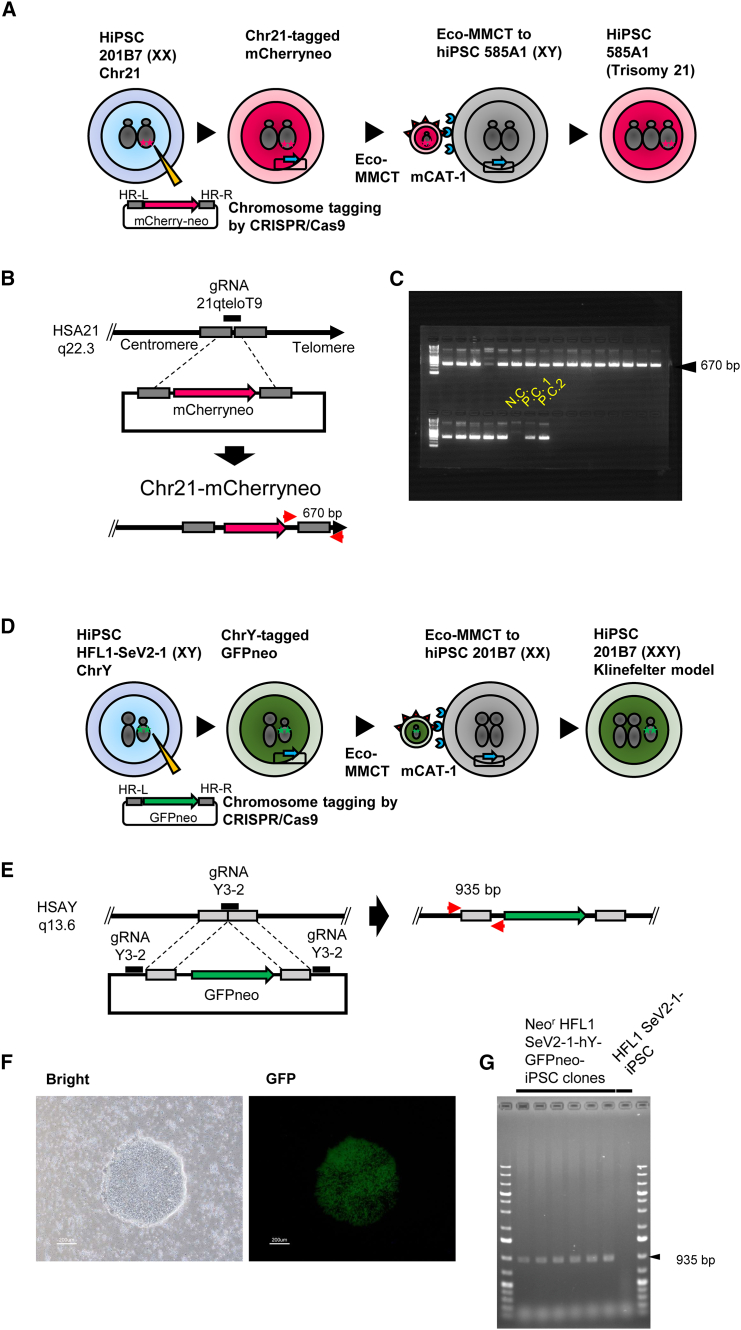
Chromosome tagging by CRISPR-Cas9 for MMCT of targeted native Chr21 and Y (A) Schematic illustration of MMCT following chromosome tagging for Chr21. hiPSC 201B7 with a normal karyotype 46,XX was transferred with Chr21-tagged *mCherryneo* from hiPSCs 585A1 that were 46,XY. hiPSC 201B7 transferred with Chr21 showed trisomy 21 indicating Down syndrome. (B) Schematic diagram of chromosome tagging with *mCherryneo* on Chr21 by CRISPR-Cas9 is shown. The primer set detected 670 bp of the right homologous arm region. (C) PCR analysis of 21 G418-resistant clones to detect correct gene insertion. Negative control (N.C., 201B7), positive controls (P.C. 1 and 2, bulk samples from two replicate experiments, in which 201B7 were transfected with CRISPR-Cas9 and the plasmid vector containing *mCherryneo*). (D) Schematic illustration of MMCT following chromosome tagging for ChrY (upper diagram). hiPSC 201B7 with a normal karyotype 46,XX was transferred with ChrY-tagged *GFPneo* from HFL-1-iPSC that were 46,XY. hiPSC 201B7 transferred with a ChrY was XXY, indicating Klinefelter syndrome. (E) Schematic diagram of chromosome tagging with *GFPneo* on ChrY by CRISPR-Cas9. The primer set detected 935 bp of the left homologous arm region. (F) Representative images of a neo-resistant colony observed by microscopy (left, bright phase contrast; right, GFP fluorescence). Scale bars, 200 μm (white). (G) PCR analysis of the G418-resistant clones to detect correct gene insertion.

Unlike ChrX, which carries an *HPRT* gene, there are no known endogenous drug-resistant genes applicable to MMCT of autosomes and ChrY. To obtain clones to which a chromosome had been transferred, a selectable marker gene was pre-inserted into the chromosomes. The *mCherryneo* gene was inserted into Chr21 using CRISPR-Cas9. 201B7 (XX) as a CDC and 585A1^27^ (XY) as a CRC were used to distinguish CDCs and CRCs by karyotyping (Figure 3A). The *mCherryneo*-containing plasmid vector was introduced by targeting the telomere region of Chr21 and cleaving it with CRISPR-Cas9, flanked by 300 bp of the homologous arm region (HR) (Figure 3B). Twenty-one G418-resistant clones were obtained, and PCR analysis confirmed the expected *mCherryneo* insertion in all clones (Figure 3C).

The *GFPneo* was inserted into ChrY using CRISPR-Cas9. HFL1 SeV2-1 (XY) as CDC and 201B7 (XX) as CRC were used to distinguish CDCs and CRCs by karyotyping (Figure 3D). The *GFPneo* was inserted near the *UTY*, the euchromatin region, and was expected to be consistently expressed, without regional duplication (Figure 3E). Six GFP-positive (Figure 3F) and G418-resistant clones were obtained, and PCR analysis confirmed the expected *GFPneo* insertion in all clones (Figure 3G). One of each of these CRISPR-Cas9-tagged hiPSC clones, harboring Chr21-tagged *mCherryneo* and ChrY-tagged *GFPneo,* was randomly selected to be the CDC for subsequent chromosome transfer.

### MMCT of native Chr21, ChrX, and ChrY between non-identical hiPSCs

**Figure 4:**
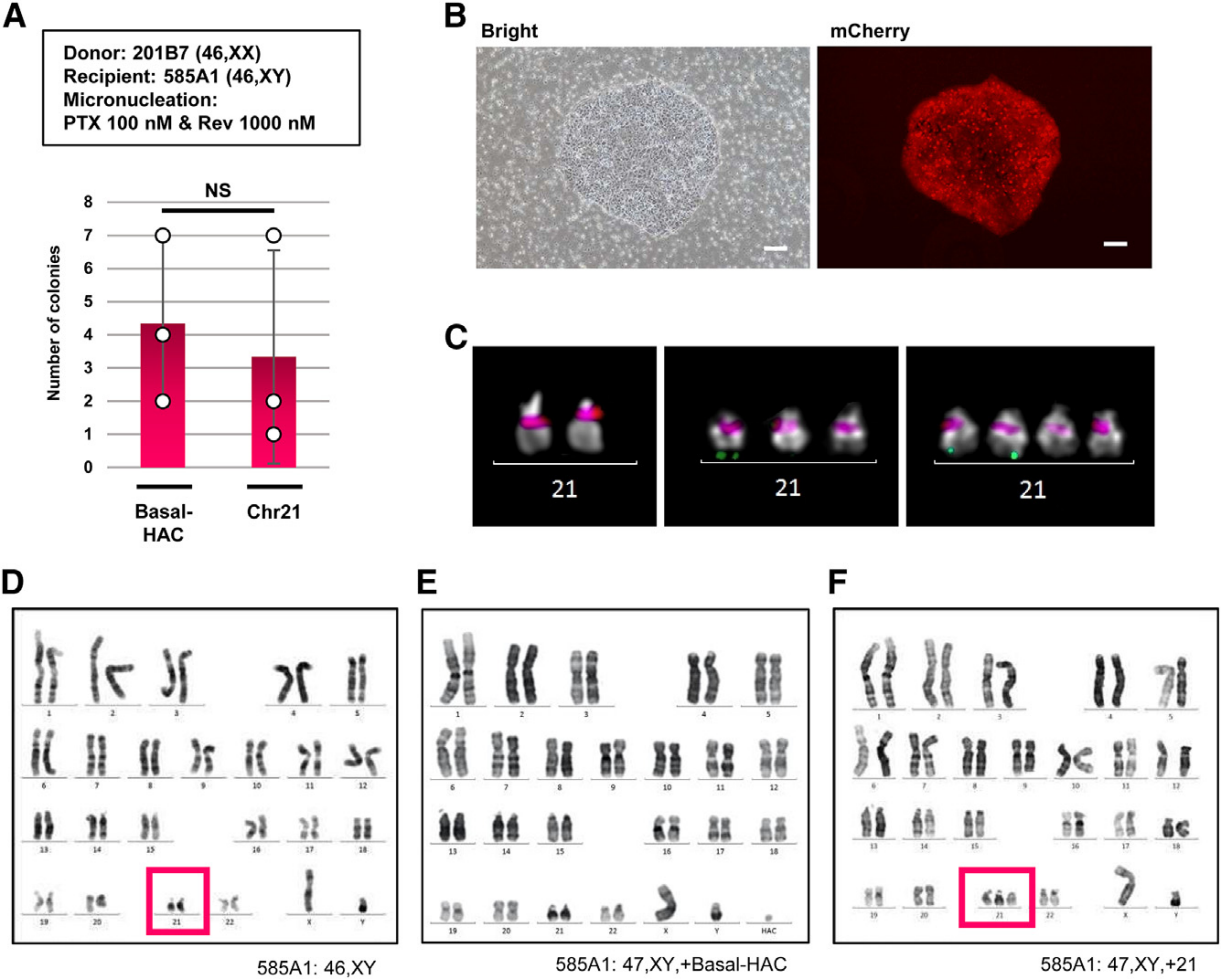
Transfer of native Chr21 using MMCT from hiPSCs to hiPSCs (A) MMCT efficiency of Basal-HAC from hiPSCs (201B7-Basal-HAC) or Chr21 from hiPSCs containing tagged-Chr21 to hiPSC 585A1. Data are the mean ± SD (*n* = 3). NS, not significant. (B) Representative images of a drug-resistant colony (left, bright phase contrast; right, mCherry fluorescence). Scale bars, 100 μm (white). (C) FISH of disomy Chr21 in 585A1 (left), trisomy Chr21 (center), and tetrasomy Chr21 in Chr21-transferred 585A1 (gray, DAPI; red, D21Z1; green, *mCherryneo*). (D–F) Quinacrine-Hoechst (QH) counter-stain karyotype images of 585A1, 585A1 Basal-HAC, and 585A1 trisomy chr21 clones. Red frames, Chr21.

We attempted the MMCT of various sizes/types of endogenous chromosomes from hiPSCs to hiPSCs. Transfer of Chr21 was performed with 201B7 (XX) containing Chr21-tagged mCherryneo to 585A1 (XY) (Figure 3A). We successfully obtained an average of 3.33 ± 3.21 (SD) G418-resistant and mCherry-expressing colonies (Figures 4A and 4B). By comparison, transferring Basal-HAC from 201B7-Basal-HAC into 585A1 yielded an average of 4.33 ± 2.52 (SD) colonies (Figure 4A), indicating a similar efficiency in the transfer of Chr21 and Basal-HAC into hiPSC 585A1. A summary of the FISH analysis of 585A1 clones into which the Basal-HAC was transferred is provided in Table S3. FISH analysis of 585A1 clones as CRCs indicated Chr21 disomy (Figure 4C, left), and the obtained clones exhibited Chr21 trisomy, one of which was tagged with mCherryneo (Figure 4C, center). Furthermore, an obtained clone exhibited Chr21 tetrasomy, two of which were tagged with mCherryneo (Figure 4C, right; Table S4). Karyotyping confirmed 585A1 as a CRC was 46,XY (Figure 4D), representative 585A1 transferred with Basal-HAC was 47,XY,+Basal-HAC (Figure 4E) and with Chr21 was 47,XY,+21 (Figure 4F). There were no notable chromosomal abnormalities in the host chromosomes, nor were there any cases of transferred chromosomes other than Chr21 from CDCs.

**Figure 5:**
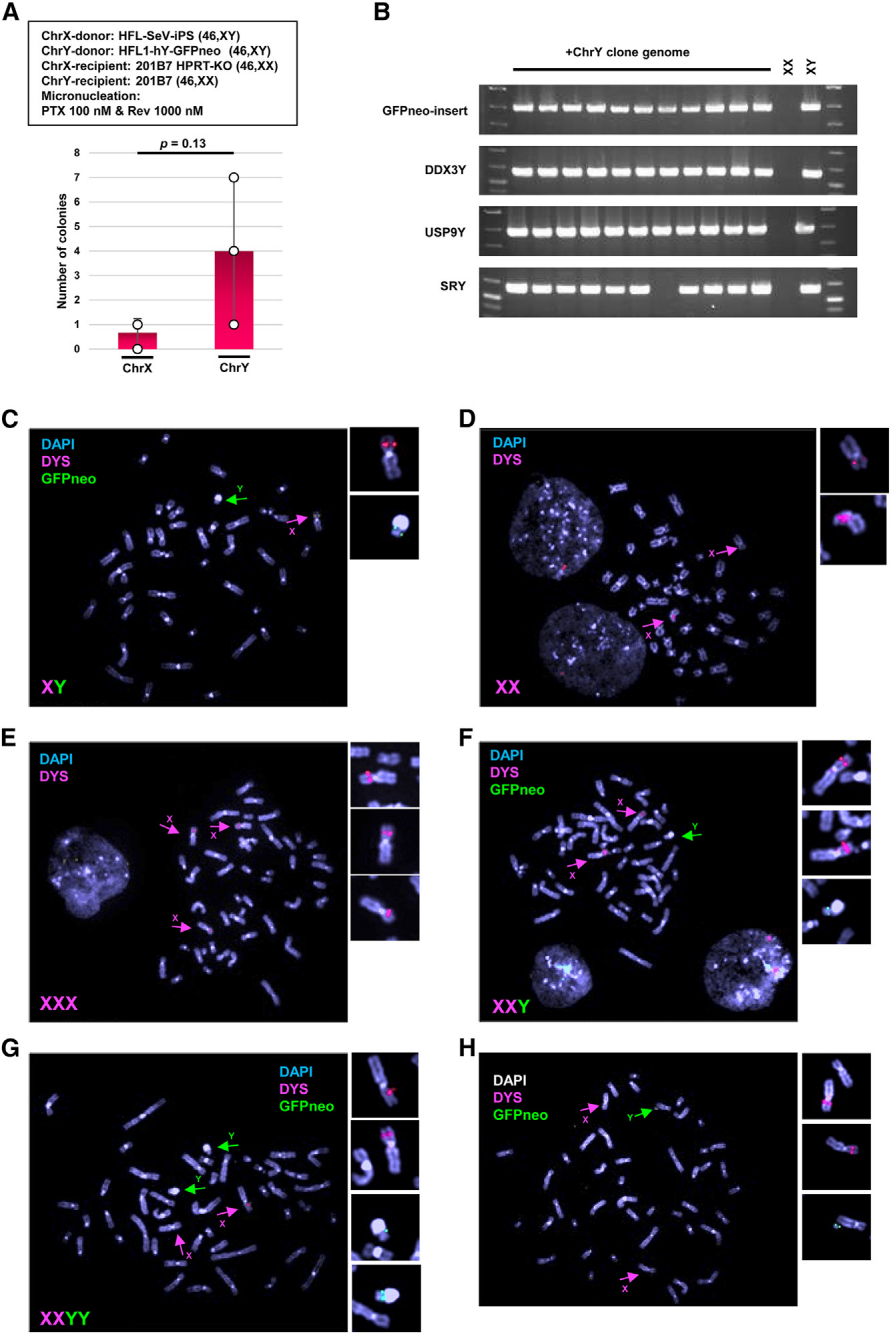
Transfer of native ChrX and ChrY using MMCT from hiPSCs to hiPSCs (A) MMCT efficiency of ChrX from 201B7 (46,XX) to 201B7 HPRT-KO (46,XX) or ChrY from HFL-1-hY-GFPneo Y to 201B7 (46,XX). Data are the mean ± SD (*n* = 3). (B) PCR analysis of ChrY-specific genes. (C) FISH analysis of HFL1-SeV-2-1 iPSC providing ChrX and ChrY (gray, DAPI; red, *dystrophin* gene (*DYS*); green, *GFPneo*). Magenta arrows, ChrX; green arrows, ChrY. Enlarged images are images of ChrX and ChrY. (D) FISH analysis of HFL1-SeV-2-1 iPSC accepting ChrX and ChrY (gray, DAPI; red, *DYS* gene). (E and F) FISH analysis of clones with trisomy X (E) and XXY (F). Light blue, DAPI; red, *DYS* gene on ChrX; green, *GFPneo* on ChrY. (G) Disomy of exo-ChrY (gray, DAPI; red, *DYS* gene; green, *GFPneo*). (H) Fragmented ChrY showing the deletion of *SRY* by PCR analysis (gray, DAPI; red, *DYS* gene; green, *GFPneo*). Magenta arrows, ChrX; green arrows, ChrY.

Next, transferring ChrX and ChrY between opposite-sex hiPSCs was attempted to determine whether the obtained clones were derived from CDCs and CRCs. When transferring ChrX from male iPSCs (HFL1-SeV-iPS, 46,XY) to the modified iPSC line (201B7, 46,XX, HPRT-KO),^23^ two HAT-resistant clones (average 1 ± 1 SD clone) were obtained (Figure 5A). When transferring ChrY from male hiPSCs (HFL1-hY-GFPneo) to female hiPSCs (201B7, 46,XX), an average of 4 ± 3 (SD) clones were obtained (Figure 5A). Eleven clones of Y-transferred 201B7 that proliferated were analyzed by PCR and the presence of ChrY was confirmed, and one clone showed disruption of the *SRY* gene (Figure 5B).

Transferred ChrX and ChrY were verified by FISH using ChrX-detecting and GFPneo probes (Figures 5C–5H) and karyotyping. Having confirmed HFL1-hY-GFPneo was 46,XY with GFPneo-tagged ChrY (Figure 5C) and 201B7 was 46,XX (Figure 5D), we generated triple X syndrome model (47,XX,+X) iPSCs (Figure 5E) by transferring ChrX between hiPSCs (Table S5), and various Klinefelter's syndrome models from female iPSCs (Table S6) that were 47,XX,+Y (Figure 5F), 48,XX,+Y + Y (Figure 5G), and 47, XX,+Ypq- (Figure 5H) by transferring ChrY from male iPSCs to female iPSCs.

### Verification of chromosome integrity

**Figure 6:**
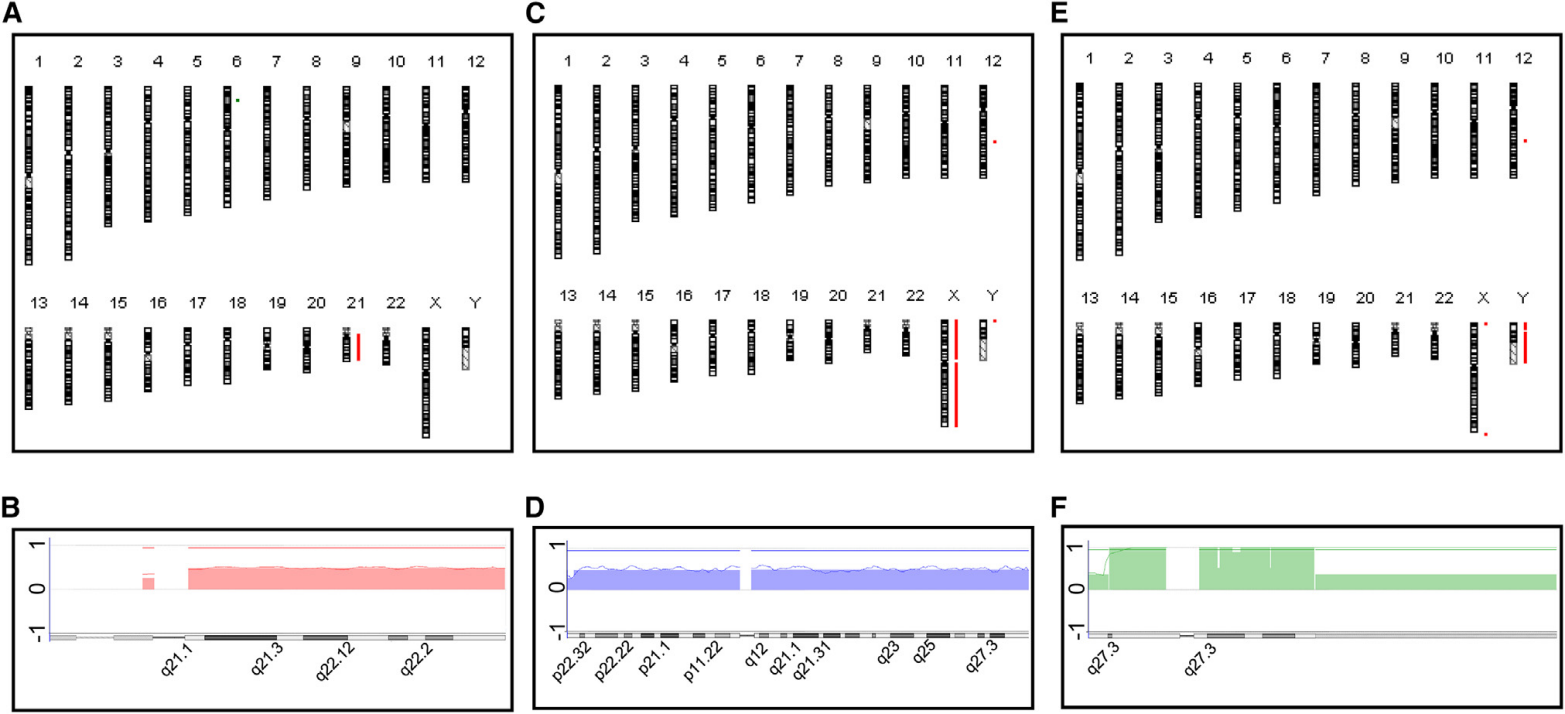
Whole-genome CGH array analysis of the representative clones transferred with Chr21, ChrY, or ChrX This figure presents the CGH array analysis of representative clones that were transferred with Chr21 (585A1-21Exp.2#01), ChrX (201B7 HPRT-KO-X Exp1-1), or ChrY (201B7-YGFPneoExp3-1). The karyotype shown includes all human chromosomes from 1 to 22, X, and Y. Red lines indicate regions of amplification, while green lines indicate regions of deletion. (A) A summary ideogram of the whole genome in the analysis of 585A1-21Exp.2#01 compared with 585A1. (B) A summary ideogram of Chr21 from the CGH Analytics software for 585A1-21Exp.2#01. (C) A summary ideogram of the whole genome in the analysis of 201B7 HPRT-KO-X Exp1-1 compared with 201B7. (D) A summary ideogram of ChrX from 201B7 HPRT-KO-X Exp1-1. (E) A summary ideogram of the whole genome in the analysis of 201B7-YGFPneoExp3-1 compared with 201B7. (F) A summary ideogram of ChrY from 201B7-YGFPneoExp3-1. The graph shows the average log2 ratios, indicating the relative copy number changes of chromosomes. A value of 1 on the y axis represents a 2-fold increase in chromosome number and a value of −1 indicates a halving of the chromosome number (A–E).

During the MMCT process, there was a concern that chromosomal amplifications or deletions might occur. To verify that the transferred chromosomes were intact and maintained their integrity, whole-genome comparative genomic hybridization (CGH) microarray analysis was conducted to quantitatively assess the comprehensive chromosome copy number, including the introduced chromosomes, for clones with introduced Chr21, ChrX, and ChrY. Based on the results of Q-banding karyotype analysis, we randomly selected a representative clone with trisomy 21 from those with an ideal karyotype (585A1-21Exp.2#01) (Table S4). For the triple X syndrome model iPSCs, we selected one clone (201B7HPRT-KO-X Exp1-1) that exhibited 47,XX,+X at a high ratio (Table S5). For the Klinefelter syndrome model iPSCs, we randomly selected a representative clone (201B7-YGFPneoExp3-1) that exhibited 47,XX,+Y at a high ratio (Table S6). The results of these CGH analyses indicated that there were no notable chromosomal deletions or amplifications in the host chromosomes and the transferred Chr21 (Figures 6A and 6B; Table S7), ChrX (Figures 6C and 6D; Table S8), and ChrY (Figures 6E and 6F; Table S9) in each clone. Although copy number abnormalities were observed in the 12q21.2 region of CRC by the whole-genome analysis (Figures 6C and 6E), no common abnormalities were found across all analyzed clones. All data of the CGH array analysis are provided in Table S10.

### Karyotype stability of aneuploid cells with the transferred chromosomes in long-term cell cultures

**Figure 7:**
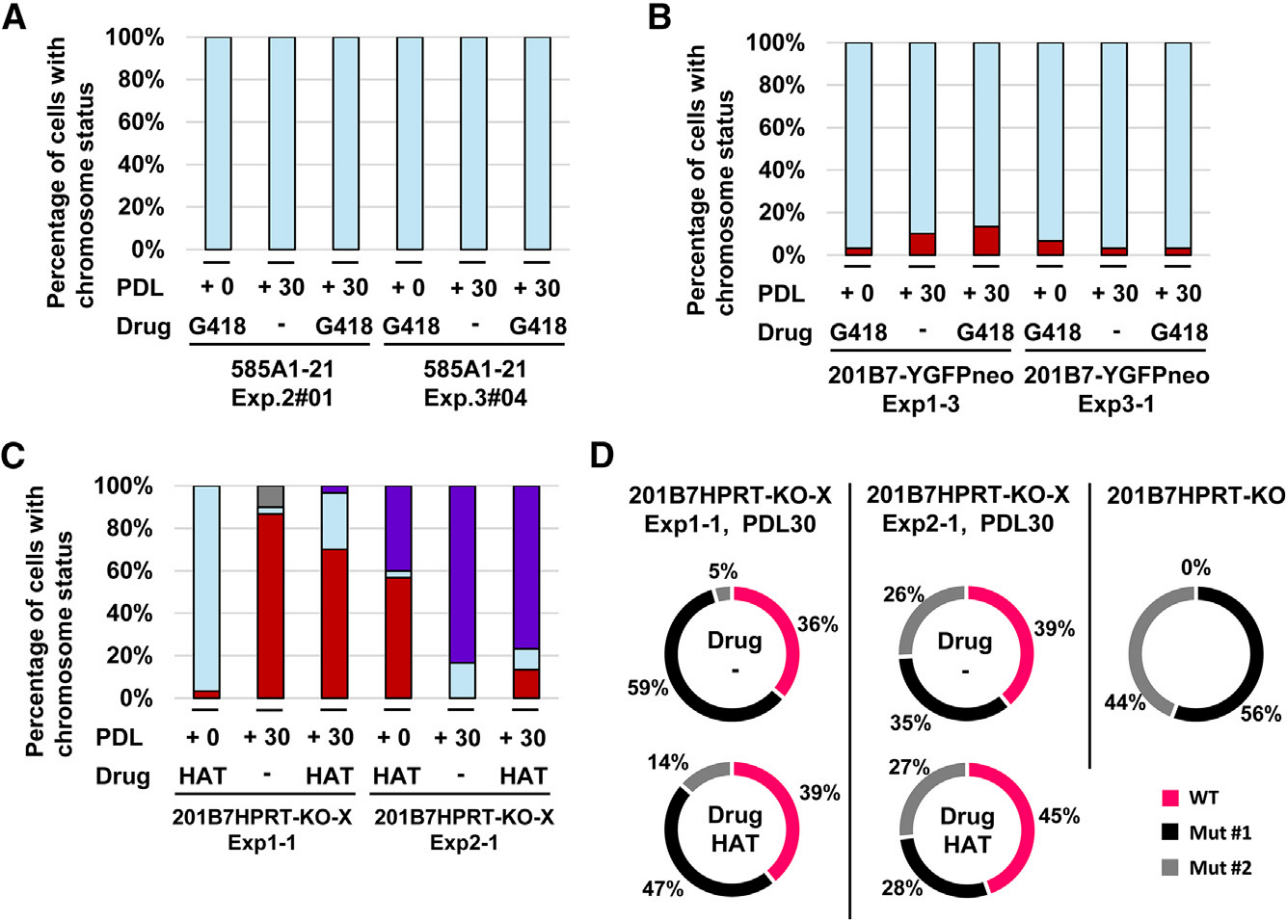
Stability of transferred chromosomes and comparison of cell proliferation in long-term cultures (A) Percentage of cells with the three Chr21 copies in Chr21-transferred clones (585A1-21 Exp.2#01 and 585A1-21 Exp.3#04) at PDL 0 and PDL 30, with and without drug selection (*n* = 30 cells for each condition). (B) Percentage of cells with ChrY in ChrY-transferred clones (201B7-YGFPneo Exp.1-3 and 201B7-YGFPneo Exp.3-1) at PDL 0 and PDL 30, with and without drug selection. The red indicates the proportion of cells that do not retain the ChrY (*n* = 30 cells for each condition). (C) Percentage of cells with different karyotypes (48,XX,+X,+X; 47,XX,+X; 46,XX) in ChrX-transferred clones (201B7 HPRT-KO-X Exp.1-1 and 201B7 HPRT-KO-X Exp.2-1) at PDL 0 and PDL 30, with and without drug selection. Light blue indicates the proportion of cells retaining the transferred chromosome, gray indicates the proportion of cells with ChrX translocations, and indigo indicates the proportion of cells with four ChrX copies (*n* = 30 cells for each condition) (A–C). (D) Ratios of wild-type (WT) and mutant alleles (Mut #1 and Mut #2) of the *HPRT* gene in 201B7HPRT-KO-X Exp1-1, 201B7HPRT-KO-X Exp2-1, and 201B7HPRT-KO cell lines at PDL 30, with and without drug selection. WT is indicated in pink, Mut #1 in black, and Mut #2 in gray.

We analyzed the long-term stability of the transferred chromosomes (Chr21, ChrY, and ChrX) in CRCs under conditions with and without drug selection. Two clones for each chromosome (Tables S4–S6) were cultured for approximately 30–50 days, with passaging every 5 days until a population doubling level (PDL) of 30 was reached. Karyotype analysis was performed using Q-banding (Figures S2–S4). For each condition, the number of transferred Chr21 (Figure 7A), ChrY (Figure 7B), and ChrX (Figure 7C) retained was examined in 30 cells. Chr21-transferred iPSC clones maintained a population with 100% of the cells retaining the 47,XX,+21 karyotype, regardless of drug selection (Figure 7A and S2A–S2F). ChrY-transferred clones showed high ChrY retention rates of 83%–97%, with no observable effect of drug selection on retention rates (Figure 7B and S3A–S3F). In contrast, ChrX-transferred cells exhibited clone-specific results. 201B7 HPRT-KO-X Exp1-1 showed progressive ChrX loss over passages, with a slightly higher tendency for loss under non-selective conditions (Figure 7C and S4A–S4F). 201B7 HPRT-KO-X Exp2-1 was initially comprised of 40% cells with 48,XX,+X,+X, 3% with 47,XX,+X, and 57% with 46,XX karyotypes. The proportion of cells with 48,XX,+X,+X increased over time, reaching 77% without selection and 83% with selection at PDL+30. To assess the stability of the transferred ChrX following the observation of ChrX loss or amplification after long-term culture, we evaluated the ratio of exogenous to endogenous chromosome alleles, followed by NGS analysis with an amplicon to quantify the ratio of wild-type (WT) alleles to two types of mutant alleles of the *HPRT* gene (Figure 7D).We analyzed the two ChrX-transferred clones and the parent 201B7 HPRT-KO cell line, which had a disrupted *HPRT* gene that caused specific mutations in each endogenous ChrX. The transferred ChrX retained the WT allele, allowing distinction among the three alleles in the ChrX-transferred clones. The sequences of the mutant alleles in the *HPRT* gene were determined by Sanger sequencing (Table S11). For 201B7HPRT-KO-X Exp1-1, disomy related to ChrX loss was observed after long-term culture, regardless of drug selection, with WT allele retention of 36%–39%. In contrast, mutant allele #2 had a retention rate of 5%–14%, suggesting a notable contribution to disomy. For 201B7HPRT-KO-X Exp2-1, the equal presence of two mutant alleles and one WT allele in tetrasomic cells after long-term culture suggests that the WT allele was amplified to two copies, whereas the remaining two alleles were mutant alleles. Cell proliferation rates were compared among clones and culture conditions (Figure S5). Despite the observed tendency for growth suppression under HAT selection, no significant differences were found between the 201B7HPRT-KO-X Exp1-1 and 201B7HPRT-KO-X Exp2-1 clones under HAT selection or non-selection conditions. This indicated that there was no apparent relationship between cell proliferation capacity and ChrX copy number (Figures 7C, 7D, and S5). ChrY and ChrX-transferred clones showed no significant differences in growth rates with or without drug selection. Taken together, these data suggest that transferred chromosomes were stably maintained in the CRCs.

### Assessment of capability of trilineage differentiation of the chromosome transferred aneuploid hiPSCs

To verify whether pluripotency was maintained after chromosome transfer via MMCT, we performed a teratoma formation assay for hiPSC clones obtained through the transfer of Chr21, ChrX, and ChrY (585A1-21Exp.3#04, 201B7 HPRT-KO-X Exp1-1, 201B7-YGFPneoExp3-1). The hiPSC clones were transplanted into the testes of immunodeficient mice and teratoma formation was observed. Histological analysis was performed on the resulting teratomas. Melanin-producing tissues, cartilage, and glandular structures were observed as indicators of ectodermal, mesodermal, and endodermal differentiation, respectively. Differentiation into all three germ layers was confirmed for each clone (Figure S6). These results indicate that all clones exhibited trilineage differentiation, demonstrating that pluripotency was maintained even after chromosome transfer.

## Discussion

**Figure 8:**
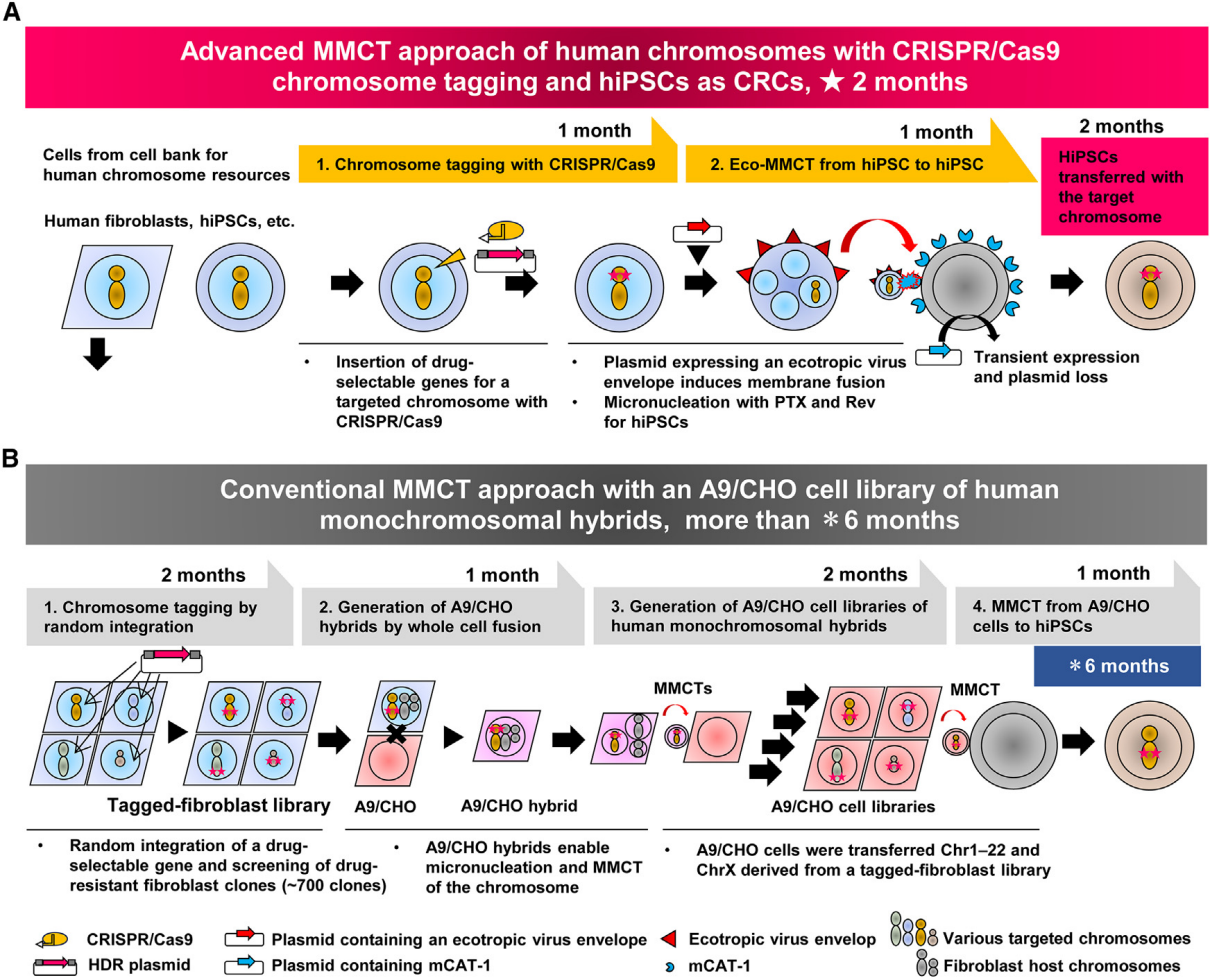
Schematic illustration of the advanced MMCT approach for human chromosomes using CRISPR-Cas9 chromosome tagging in hiPSCs compared with the A9/CHO cell library approach (A) The advanced MMCT is a straightforward approach for transferring a targeted chromosome into hiPSCs. First, a drug-selectable gene is inserted into the target chromosome using CRISPR-Cas9 through homologous recombination. For hiPSCs, an ecotropic virus envelope gene, which induces membrane fusion, is introduced into the CDCs, and the receptor gene (mCAT-1) is transiently introduced into the CRCs. PTX and Rev are used to induce micronucleation in the CDC. Microcells with the ecotropic virus envelope on their membrane can be obtained from this CDC, allowing chromosome transfer into the CRC. Then, the chromosome can be transferred to hiPSCs via MMCT. In this approach, hiPSCs with the introduced target chromosome can be established through the two-step chromosome and cell manipulation process. This advanced method reduces the overall process time to 2 months. (B) In the conventional MMCT approach using an A9/CHO cell library, the chromosome in human fibroblasts, which act as a chromosome resource, is tagged with a drug-selectable gene using the random integration method. The resulting drug-resistant clones are screened, and clones tagged on the target chromosome are selected. Next, whole-cell fusion is performed with A9/CHO cells to obtain A9/CHO hybrids containing the tagged target chromosome. To separate the target chromosome from the non-target human chromosomes derived from human fibroblasts, the target chromosome is introduced into the A9/CHO cells by MMCT. This results in the A9/CHO cell libraries of human monochromosomal hybrids. Finally, chromosomes from the A9/CHO cell libraries are introduced into hiPSCs using MMCT. In this approach, hiPSCs with the introduced target chromosome can be established through the four-step chromosome and cell manipulation process. The conventional method requires over 6 months to complete.

This study demonstrated the efficient chromosome transfer from hiPSCs with PTX and Rev into model human cell line HT1080 (Figure 1C). Then, we developed an advanced MMCT that enabled the systematic transfer of chromosomes from hiPSC to hiPSC, significantly reducing the experimental period (Figures 8A and 8B) and achieving the generation of isogenic aneuploidy models. These models were generated using CRISPR-Cas9 chromosome tagging, the transient expression of an ecotropic virus envelope and mCAT-1 (Figure 1A). The conventional A9/CHO cell library approach required four steps for completion (Figure 8B). In contrast, using the advanced MMCT method, this approach was streamlined to a more efficient two-step process (Figure 8A).

The correction of XO to XX, which is uniparental disomy of ChrX in Turner syndrome model hiPSCs, has conventionally relied on the occurrence of the uniparental duplication of ChrX during somatic cell reprogramming,^15^ but at a low frequency. Because XO could be converted to XX in HT1080 (Figure 2E), we inferred that chromosome number correction might be possible for Turner’s syndrome model hiPSCs.

Importantly, this study represents the first report of transferring an intact ChrY, tagged with drug markers, into target cells using MMCT. The transfer of ChrY fragments was reported previously,^28^ but A9/CHO cells containing ChrY tagged with a drug-resistant gene could not be established. Targeting ChrY might previously have been unsuccessful because of the inability to insert drug-resistant genes to euchromatin regions of ChrY, which comprises approximately 50% of the heterochromatin regions, using the random integration methods of the 1990s.^29^ The tagging of ChrY with CRISPR-Cas9 allows the transfer of ChrY with complex repetitive sequences,^30^ which are difficult to create artificially by DNA synthesis, into any cell for functional analysis. Furthermore, CRISPR-Cas9 chromosome tagging and MMCT from hiPSCs as CDCs might be of use for the investigation of familial inheritance of ChrX including fragile X syndrome,^9^ and inheritance of ChrY focusing on Y haplogroups.^31^

MMCT efficiency was strongly correlated with the number of CDCs used and the number of microcells derived from CDCs.^19^ Therefore, the micronucleation rate and microcell yield in hiPSCs were similar to or slightly lower than the conventional use of CHO cells as CDCs, indicating that hiPSCs can be widely used as CDCs. The additional screening of compounds with PTX- and Rev-related molecular mechanisms might improve microcell yields and the MMCT efficiency further. Regarding microcell purification using membrane filters, different filter pore diameters altered the yield of microcells retaining ChrX and MMCT efficiency, but not MAC6, Basal-HAC, Chr21, or ChrY (Figure 2A; Table S1). With reference to the results of chromosome size and micronucleus area reported previously,^27^ the median diameter of the micronucleus harboring Chr21 (chromosome size of approximately 45 Mb, micronuclei; estimated [est.] 2 mm^2^, as inferred from the graph in the reference) was estimated to be 1.6 μm (diameter = √(area/π), and the median diameter of the micronucleus harboring ChrX (chromosome size of approximately 154 Mb, micronuclei; est. 5 mm^2^, as inferred from the graph in the reference) was estimated to be 2.5 μm. The micronuclei containing ChrX may include micronuclei with diameters larger than 3 μm.^27^ This could be interpreted as an increase in the yield of large microcells and improved MMCT efficiency of ChrX when using 5-μm pore diameter filter filtration rather than 3-μm pore diameter filter filtration. However, the MMCT efficiency for ChrX remained relatively low compared with ChrY, and was insufficient when using the 5-μm pore diameter filter purification method (Figure 5A).

Using the new MMCT method, we transferred chromosomes from hiPSCs to hiPSCs and found that entire chromosomes could be transferred regardless of their length (Figures 6A–6F). Previous studies using A9/CHO libraries and conventional MMCT methods reported chromosome deletions and structural abnormalities in the introduced chromosomes related to structural abnormalities in the donor cells and chromosome damage in micronuclei.^10^^,^^32^ The generation of mouse/rat models retaining fragments of chromosome 21 using A9/CHO libraries also showed partial deletions in the introduced chromosome fragments.^33^^,^^34^ Moreover, a comprehensive analysis was not performed for trisomy hPSC model cells with human chromosomes 8, 13, 18, and 21 generated by A9 libraries.^6^^,^^7^ In a study where ChrX was transferred into hiPSCs via A9/CHO cells inducing micronucleation with colcemid,^12^ it was speculated that the full-length ChrX was introduced based on whole-exome sequencing, but reproducibility, non-coding regions, and other chromosomes were not verified. Furthermore, a comprehensive analysis of chromosome integrity using the MMCT method with PTX and Rev, a spindle stabilizer, and spindle assembly checkpoint inhibitor, was not performed.^19^^,^^20^ The MMCT method developed in this study suggests that complete chromosomes with normal structures can be introduced. In addition, even chromosomes such as Chr21^35^ and ChrX (Figure 7C) that undergo spontaneous loss in hiPSCs, can be introduced by directly transferring chromosomes from normal hiPSCs. After transferring ChrX, hiPSC clones lost one ChrX or gained an additional ChrX during passaging (Figure 7C), likely due to selective growth advantages. NGS analysis showed stable maintenance of the transferred ChrX (Figure 7D). Changes in ChrX copy number might be driven by the slight proliferative advantages of the transferred ChrX and the growth-suppressive effect of the Mut #2 allele, rather than *HPRT* gene copy number and drug resistance, and the fate of these changes appears to be determined for each clone immediately after chromosome transfer (Figures 7C, 7D, and S5). The different dynamics of the ChrX indicated changes in proliferation control factors on ChrX, including X inactivation erosion in hiPSCs.^36^ To maintain trisomy X karyotype cells, improved culturing or enrichment methods through cloning and other techniques are necessary.^37^

In the future, the advanced MMCT approach using hiPSCs as CDCs and CRCs offers significant potential for studying rare diseases and early embryonic development. This method allows for the generation of isogenic hyperaneuploidy hiPSCs, akin to the establishment of hiPSCs derived from patients with Down syndrome, Klinefelter’s syndrome, and triple X syndrome, as reported previously.^8^^,^^16^^,^^17^ By targeting non-lethal transferred chromosomes, this method facilitates the transfer of a comprehensive set of chromosomes or regions known to influence early development into hiPSCs. Consequently, this approach might uncover novel mechanisms of early developmental processes mediated by hyperaneuploidy, thereby advancing our understanding of human developmental biology and genetic disorders.

## Materials and methods

### Cell culture

All hiPSCs were maintained in StemFit AK02N (Takara Bio, Kusatsu, Japan) and iMatrix-511 Silk (Takara Bio) following the manufacturer’s instructions. The iPSC lines 201B7 (HPS0063)^14^ and 585A1 (HPS0354)^27^ were purchased from Riken BioResource Research Center (Tsukuba, Japan). The generation of 201B7-MAC6 was reported previously.^23^ The cell line 201B7-MAC6 was cultured with StemFit AK02N containing 90 μg/mL of G418 (Fujifilm Wako, Chuo-ku, Osaka, Japan). *HPRT* gene KO 201B7 cells were reported previously.^23^ The hiPSC line 201B7-Basal-HAC^7^ was generated by the transfer of Basal-HAC from hiPSCs as previously reported.^7^ HFL1-iPS (HFL1 SeV2-1) cells,^7^ derived from the reprogramming of human fetal lung fibroblasts HFL-1 (RCB0521), were established using a SeV vector system^20^ (ID Pharma, Tsukuba, Japan) and HFL1-HAC-iPSC,^26^ as described previously. HT1080 (CCL-121) were purchased from the American Type Culture Collection (Manassas, VA) and maintained in Dulbecco’s modified Eagle’s medium (DMEM) (Fujifilm Wako) supplemented with 10% fetal bovine serum (Sigma-Aldrich, Saint Louis, MO) and 1% penicillin/streptomycin (Fujifilm Wako). *HPRT* gene KO HT1080 cells were reported previously.^23^

### Gene transfection by electroporation for the expression of mCAT-1 toward Eco-MMCT

Plasmid sequences expressing mCAT-1 (pEF1-mCAT-1) are described in Table S5. The primers (Table S6) mCat1fw1 and mCat1rv2 amplified mCAT-1 cDNA derived from NIH3T3 cells. The amplicon generated by mCat1 inf fw2 and mCat1 inf rv2 was cloned using an In-Fusion HD Cloning kit (Takara Bio) to *Eco*RI- and *Not*I-digested pEF1-GFP^38^ (Addgene, plasmid no. 11154; http://n2t.net/addgene:11154; RRID: Addgene_11154). The plasmid, pEF1-mCAT-1, was transfected into HT1080 by lipofection and electroporated into HT1080 and hiPSCs. The plasmids were purified with NucleoBond Xtra Midi (Macherey-Nagel, Düren, Germany) following the manufacturer’s instructions. Lipofection was performed using Lipofectamine LTX Reagent (Thermo Fisher Scientific, Waltham, MA) following the manufacturer’s protocol. The Super Electroporator NEPA21 (Nepa Gene, Ichikawa, Japan) was used to introduce plasmids into HT1080 or hiPSCs. Cells (2 × 10^6^) were prepared in 100 μL Opti-MEM (Thermo Fisher Scientific) with 20 μg plasmid DNA in a 2-mm cuvette (Nepa Gene). Poring pulse conditions were 175 V, 2.5 ms pulse length; 201B7, HFL1-SeV-iPSC, 125 V, 5.0 ms pulse length; HFL1-HAC-iPSC, 585A1, 50 ms pulse interval, two pulses, 10% attenuation rate, and (+) polarity. Transfer pulses were 20 V, 50 ms pulse length, 50 ms pulse interval, five pulses, 40% attenuation rate, and (+/−) polarity.

### FCM analysis

Cells expressing mCAT-1 were prepared (1 × 10^6^ cells) and stained to detect mCAT-1. Anti-mouse Slc7a1-conjugated APC (catalog no. 150505, BioLegend, San Diego, CA) was used following the manufacturer’s instructions. The stained cells were analyzed by FCM using a Gallios system (Beckman Coulter, Brea, CA). Generated FCS files were analyzed using Kaluza software (Beckman Coulter).

### Chromosome tagging by CRISPR-Cas9

The plasmid sequences for gene targeting were synthesized by GeneArt (Thermo Fisher Scientific) and Alt-R CRISPR-Cas9 crRNA, Alt-R CRISPR-Cas9 tracrRNA, and Alt-R S.p. HiFi Cas9 Nuclease V3 were purchased from Integrated DNA Technologies (Coralville, IA), and the targeted sequences are described in Table S10. crRNA and tracrRNA reagents were prepared at 200 μM with Nuclease-Free Duplex Buffer (Integrated DNA Technologies). A mixture of 3 μL each of crRNA and tracrRNA was prepared. Then, 6 μL of the mixture was heated at 95°C for 5 min and then allowed to cool to room temperature. This mixture of RNA complexes was combined with 8.5 μL of S.p. HiFi Cas9 (61 μM) and 10.5 μL of PBS (Fujifilm Wako). This combined solution was left undisturbed for 15 min at room temperature to allow the formation of RNP complexes. To target the mCherryneo vector to chromosome 21 (Chr21), a combination of 2 × 10^6^ 201B7 cells, RNP complex, 5 μg of the targeting vector, 5 μL of Alt-R Cas9 Electroporation Enhancer (100 μM) (Integrated DNA Technologies), and 90 μL of Opti-MEM was prepared in a 2-mm cuvette of the electroporator. Poring pulse conditions were 150 V, 5 ms pulse length, 50 ms pulse interval, two pulses, 10% attenuation rate, and (+) polarity. Transfer pulses were 20 V, 50 ms pulse length, 50 ms pulse interval, five pulses, 40% attenuation rate, and (+/−) polarity. The transduced cells were expanded for 1 week. The mCherry-expressing cells were sorted by FCM (BD FACSMelody Cell Sorter, Becton Dickinson, Franklin Lakes, NJ). The data were analyzed using the attached software BD Chorus (Becton Dickinson). The obtained mCherry-expressing cells were cultured with medium containing 90 μg/mL of G418. To target the GFPneo vector to ChrY, the cells were resuspended in StemFit AK02N medium supplemented with 10 μM Y-27632 (Fujifilm Wako) and cell counting was performed. After centrifugation, 1 × 10^6^ cells were gently resuspended in 90 μL P3 Primary Cell Nucleofector Solution from a P3 Primary Cell 4D-Nucleofector X Kit (Lonza, Basel, Switzerland). The cells were gently mixed after adding 3 μg targeting vector (pMA_RQ hY_LA-I_EGFP_I-PGKneo-5′HPRTloxP-RA), 1 μg sgRNA vector (pHL-H1 humanY3-sgRNA2-mEF1a-RiH; constructed by Addgene, no. 60601 [http://n2t.net/addgene:60601; RRID: Addgene_60601])^39^ as well as 1 μg Cas9 vector (pHL-EF1a SphcCas9-iP-A; Addgene, no. 60599; http://n2t.net/addgene:60599; RRID: Addgene_60599)^39^ and then transferred to 100 μL Nucleocuvette vessels. Immediately after electroporation using the CA-137 protocol of 4D-Nucleofector (Lonza), the cells were transferred to five iMatrix-511-coated 10-cm dishes containing 8 mL prewarmed StemFit AK02N medium with 10 μM Y-27632. At 24 h after transfection, drug-resistant cells were selected with 90 μg/mL G418 sulfate. The detailed sequences of the plasmids are described in Table S5.

### Assessment of micronucleation efficiency

Recently, we developed a novel MMCT method using hiPSCs as CDCs using PTX and Rev.^20^ Based on the results, we quantified the micronucleation efficiency of hiPSCs in detail and confirmed whether chromosome transfer between hiPSCs was feasible. The cells were incubated for 24 or 48 h to induce micronuclei in cell culture medium containing colcemid (demecolcine) (Fujifilm Wako), MAS (Genial Helix, Chester, Cheshire, UK), PTX (Fujifilm Wako), and Rev (Cayman Chemical Company, Ann Arbor, MI). Then, the cells were fixed with Carnoy’s solution (1:3 acetic acid/methanol) (Fujifilm Wako) and spread on glass slides (Matsunami Glass, Kishiwada, Japan). Nuclei were stained with 4′,6-diamidino-2-phenylindole (DAPI: 1.0 μg/mL; Sigma-Aldrich) or 5% Giemsa stain (Fujifilm Wako) to assess the micronucleation rate. Images were captured using an Axio Imager Z2 fluorescence microscope (Carl Zeiss, Jena, Germany), and the number of micronuclei per cell was analyzed with the ISIS software program (MetaSystems, Altlussheim, Germany). One hundred cells were assessed for each treatment, and the number of micronuclei was counted in each cell.

### PCR analysis

Genomic DNA was purified using a Gentra Puregene kit (QIAGEN, Venlo, the Netherlands), and genomic PCR was performed with a KOD One PCR Master Mix kit (TOYOBO, Kita-ku, Osaka, Japan), following the manufacturer’s instructions. The PCR products from the analysis of clones used for gene targeting and chromosome transfer were detected by electrophoresis with 0.8% or 2% agarose S (Fujifilm Wako) gels. PCR products using primers (Table S7) to analyze STS markers were detected by electrophoresis with 4% agarose (Agarose KANTO HC for low molecular size) (KANTO, Tokyo, Japan) gels with 1× TAE buffer. The primers (Table S6) were searched for in the UCSC Genome browser. GeneRuler 1kb Plus DNA Ladder (Thermo Fisher Scientific) was used as a molecular size marker.

### FISH analysis and Q-banding using quinacrine and Hoechst staining for chromosome analysis

For chromosome Q-banding analysis and FISH, chromosome spreads on glass slides were prepared as follows. HT1080 were treated with 0.1 μg/mL colcemid for 1.5 h, and the hiPSCs were treated with MAS (1 μg/mL in culture medium) and Chromosome Resolution Additive (Genial Helix) to induce metaphase arrest. After incubation for 1 h, the arrested cells were treated with 0.075 M KCl (Fujifilm Wako), fixed with Carnoy’s solution, and spread onto glass slides. The cells were stained with quinacrine mustard (Merck KGaA, Darmstadt, Germany) and Hoechst 33258 (Merck KGaA) to enumerate chromosomes. Images were captured with an Axio Imager Z2 fluorescence microscope and analyzed with ISIS or Ikaros software (Carl Zeiss, Darmstadt, Germany). FISH analyses were performed on the prepared chromosome spreads using digoxigenin-labeled mouse Cot-1 DNA (Merck KGaA) to detect MAC6,^23^ digoxigenin-labeled BAC containing genomic DNA derived from ChrX (RP11-954B16), digested with *Bam*HI and *Eco*RI, to detect ChrX. Biotin-labeled targeting vectors were used to detect Chr21 and ChrY. Digoxigenin-labeled probes were detected with rhodamine-conjugated anti-digoxigenin Fab fragments (Merck KGaA). The biotin-labeled probe was detected with fluorescein isothiocyanate-conjugated avidin (Merck KGaA). The biotin and digoxigenin labeling of DNA was performed using a Nick Translation Mix (Roche, Basel, Switzerland). Chromosomal DNA was counterstained with DAPI (Fujifilm Wako). Images were captured using an Axio Imager Z2 fluorescence microscope and analyzed with ISIS software. mFISH analyses were performed in accordance with the manufacturer’s instructions (MetaSystems).

### Assessment of MMCT efficiency from hiPSCs

hiPSCs expressing an ecotropic virus envelope as CDCs were prepared in a 10-cm dish until confluency was reached. For each experiment, 1.2 × 10^6^ CDCs were prepared. The cells were then detached, counted, and aliquoted at 2 × 10^6^ cells per iMatrix-511 silk, overnight coated, TC-25 flask (Thermo Fisher Scientific). To each flask, 4 mL of 10 μM Y-27632 in AK02 medium and iMatrix-511 silk were added following the manufacturer’s instructions. On the next day, the medium was replaced with AK02 containing PTX and Rev, and the cells were cultured for 48 h at 37°C. Subsequently, cells were centrifuged to acquire microcells. The medium was removed, and each flask was filled with approximately 40 mL of DMEM containing 10 μg/mL cytochalasin B (Sigma-Aldrich). The flasks were then centrifuged at 12,000 × *g* for 60 min at 37°C. The supernatant was discarded, and the pellet of microcells was resuspended in 2 mL of serum-free DMEM per flask. The suspension involving microcells was collected and resuspended five times using a 20G syringe gauge and homogenized. The suspension was then sequentially filtered through 8- and 5-μm Nucleopore Polycarbonate Track Etch Membrane filters (Cytiva, Shinjuku-ku, Tokyo, Japan), and twice through 3-μm filters (with/without 3-μm filters in Figure 2A). The filtered suspension was centrifuged at 760 × *g* for 10 min to collect the microcells. Then, the microcells were resuspended in 4 mL of culture medium and co-cultured with 1 × 10^6^ HT1080 cells or hiPSCs expressing mCAT-1 in a 6-cm dish in triplicate. After 24 h, the cells were passaged to three 10-cm dishes. The medium was replaced with selection medium 24 h later. Then, optimal drug selection was performed and the culture medium was changed every 3 days to obtain colonies. For PEG-MMCT, the same number of cells that did not express ecotropic virus envelope was used. The collected microcells were suspended in a solution of DMEM containing 50 μg/mL phytohemagglutinin P (Sigma-Aldrich), and the microcell suspension replaced the culture medium of the recipient cells, which were then left undisturbed at 37°C for 20 min. Next, the microcell suspension was carefully removed, and a solution of PEG1500 (Roche) containing 10% DMSO HybriMax (Sigma-Aldrich) was added, followed by incubation for 90 s. After microcell fusion induced by 90-s PEG exposure, the cells were washed three times with 5 mL of serum-free DMEM. Finally, the medium was replaced and the cells were left undisturbed at 37°C. After 24 h, the cells were passaged in the same manner as for Eco-MMCT.

### Whole-genome CGH microarray analysis

Each amplified sample (250 ng) was labeled using a SureTag Complete DNA Labeling Kit (Agilent Technologies, Santa Clara, CA). In brief, Cy3- and Cy5-labeled DNA were combined with Cot-1 DNA (Thermo Fisher Scientific) and CGH blocking agent (Agilent Technologies), then denatured and hybridized to the arrays (SurePrint G3 Human CGH Microarray 8 × 60K, Agilent Technologies) for 24 h in a rotating oven at 67°C and 20 rpm (Agilent Technologies). After hybridization and washing, the microarray was scanned using an Agilent SureScan Microarray Scanner System (G2600D). Images were analyzed with Feature Extraction Software 12.1.1.1 (Agilent Technologies), with CGH_1201_Sep17 protocol for background subtraction and normalization. Statistical analysis for whole-genome CGH microarray analysis is described below. Data analysis of the microarray experiments was conducted using the Aberration Detection Method-2 statistical algorithm (Agilent Technologies) on the basis of the combined log2 ratios at a threshold of 6.0 as described previously.^36^ Data calls with average log2 ratios <0.25 were filtered to exclude false positives.

### Long-term cell culture to evaluate chromosome stability

The evaluated iPSC clones (Tables S4–S6) were passaged and cultured under specific conditions. The long-term culture of iPSC clones began at passage day zero (PDL +0). Each clone was subjected to two conditions: with or without the addition of selection drugs at the specified concentrations described below. For clones with transferred Chr21 or ChrY, G418 was added to the culture at a concentration of 50 μg/mL. Clones with transferred ChrX were cultured in 0.2% HAT medium. Cells were seeded at a density of 1 × 10^5^ cells per well in 6-well plates, using StemFit AK02 medium at 3 mL per well. The substrate iMatrix-511 silk was added at a concentration of 1.0 μg/cm^2^. Y-27632 was added to the medium at a final concentration of 10 μM, and the cells were cultured overnight at 37°C. The following day, the medium was replaced with Y-27632-free medium containing the designated selection drugs. On the fifth day of culture, cells were detached and collected. The number of viable cells was counted using Trypan blue (Thermo Fisher Scientific) staining and measured on a Countess II FL machine (Thermo Fisher Scientific). This process was repeated until the cells reached a PDL exceeding 30. The PDL was calculated using the following formula: PDL = $\frac{\log\;10\left(\frac{N}{N_{0}}\right)}{\log\;10\left(2\right)}$, where *N* is the number of cells collected and $N_{0}$ is the number of cells seeded. The PDL values at each passage were compiled to evaluate cell proliferation under both conditions (with or without drug selection) for each clone. During this passaging period, beeswarm box plots comparing cell proliferation rates were generated for 6 to 9 passages. One passage that did not have a 5-day interval during this period was excluded from the analysis.

### Determination of sequences of mutated alleles of the *HPRT* gene

The 201B7-HPRT-KO cell line was generated with FokI-dCas9 to cut multiple targeted sequences, as described previously.^23^ The mutated region was amplified by the PCR primer set, HPRT sgRNA 01F and HPRT sgRNA 01R (Table S13), and TA cloning was performed with a TOPO TA Cloning Kit for Sequencing (Thermo Fisher Scientific) following the manufacturer’s instructions. Sanger sequencing was then performed by a commercial vendor (Eurofins Genomics, Ota-ku, Tokyo, Japan). Two types of deletions were observed in the 201B7 HPRT-KO cell line (Table S11). Mutated allele #1 showed a 48-bp deletion and mutated allele #2 had a 3-bp deletion and a 33-bp deletion.

### NGS analysis for allele frequency assessment in trisomy X

NGS analysis was performed by a commercial vendor (Bioengineering Lab., Sagamihara, Japan). The first PCR was conducted using KOD FX Neo polymerase (TOYOBO) following the manufacturer’s instructions. The first PCR conditions were as follows: initial denaturation at 94°C for 2 min, followed by 30 cycles of denaturation at 98°C for 10 s, annealing at 57°C for 30 s, and extension at 68°C for 30 s, with a final extension at 68°C for 7 min. The primers used were HPRT_01-NGS-Fw and HPRT_01_NGS-Rv (Table S13). Following the first PCR, the PCR products were purified using VAHTS DNA Clean Beads (Vazyme, Nanjing, People’s Republic of China) at a ratio of 1.0× PCR reaction volume. The second PCR was performed using KOD FX Neo polymerase with the PCR product from the first PCR. The PCR conditions included an initial denaturation at 94°C for 2 min, followed by 30 cycles of denaturation at 98°C for 10 s, annealing at 60°C for 30 s, and extension at 68°C for 30 s, with a final extension at 68°C for 7 min. The primers used were 2ndF and 2ndR. Following the second PCR, the PCR products were purified again using VAHTS DNA Clean Bead at a ratio of 1.0× PCR reaction volume, resulting in the final library. Sequencing was performed using the MiSeq system and the MiSeq Reagent Kit v.3 (Illumina, San Diego, CA) under the conditions of 2 × 300 bp. Data analysis was performed by the vendor. The obtained sequencing reads were processed to ensure high quality before analysis. The Fastx toolkit (v0.0.14) was used for the initial quality filtering. Specifically, the fastq_barcode_splitter tool extracted sequences that exactly matched the primers at the beginning of the reads. Following this, sickle (v1.33) was used to remove any reads with a quality score below 20. In addition, sequences that were reduced to 40 bases or shorter, along with their paired reads, were discarded to ensure only high-quality data were retained. Subsequent to quality filtering, high-quality paired-end reads were merged using the FLASH tool (v1.2.11). This process was conducted with standard parameters, which enabled the generation of longer continuous sequences from the high-quality reads. This merging step is crucial for improving the accuracy and reliability of the downstream analysis.^40^ Then, the frequencies of these alleles were determined using the analyzed sequences shown in Table S11 as references.

### Teratoma formation assay and histology to evaluate trilineage differentiation

To produce teratomas, 1 × 10^6^ cells of 585A1-21Exp.3#04 (47,XY,+21) and 201B7-YGFPneoExp3-1 or 3 × 10^6^ cells of 201B7 HPRT-KO-X Exp1-1 (Tables S4–S6) were injected into the testes of severe combined immunodeficiency mice (Charles River, Yokohama, Japan). After 9–13 weeks, resected teratomas were fixed in 20% formalin (Fujifilm Wako) and processed for paraffin sectioning (Sakura Finetek Japan, Chuo-ku, Tokyo, Japan), then stained with hematoxylin (Sakura Finetek Japan) and eosin (Sakura Finetek Japan).

### Statistical analysis

Significant difference tests of the efficiency of micronucleation and MMCT were performed using the Student’s t-test. Significant differences in cell proliferation rates were determined using Tukey’s HSD test.

## Data and code availability

The data that support the findings of this study are available from the corresponding author upon reasonable request.

## Acknowledgments

This study was approved by the Animal Care and Use Committee of Tottori University (permit nos. 23-Y-25, 23-Y-30, and 22-Y-36). All experiments were carried out in compliance with the ARRIVE guidelines. All methods were performed in accordance with the relevant guidelines and regulations. Mice were sacrificed by cervical dislocation prior to teratoma collection, and all efforts were made to minimize their suffering. We thank Drs. H. Kugoh, H. Abe, S. Satofuka, M. Hiratsuka, Y. Hiramuki, T. Ohira, and T. Moriwaki for critical discussions. This work was supported in part by 10.13039/501100001691JSPS KAKENHI grant nos. 18K15671 (to N.U.), 15K19615 (to N.U.), 23K05867 (to N.U.), and 18H06005 (to K.T.), Research Support Project for Life Science and Drug Discovery (BINDS) from 10.13039/100009619AMED under grant no. JP24ama121046 (to Y.K.), Centers for Clinical Application Research on Specific Disease/Organ (Type C) from 10.13039/100009619AMED under grant no. JP22bm1004001 (to Y.K. and K.T.), 10.13039/100009619AMED under grant no. JP24gm1610006 (to Y.K. and K.T.), 10.13039/100009619AMED under grant no. JP24bm1123038 (to Y.K. and N.U.), 10.13039/100009619AMED under grant no. JP24gm0010010 (to Y.K. and K.T.), 10.13039/100009619AMED under grant no. JP23am0401002 (to Y.K. and K.T.), Joint Research of the Exploratory Research Center on Life and Living Systems (10.13039/501100019770ExCELLS) (ExCELLS program no. 21-101), and JST CREST grant no. JPMJCR18S4, Japan (to Y.K. and K.T.). This research was partly performed at the Tottori Bio Frontier managed by Tottori Prefecture. We thank Susan Zunino, PhD, and J. Ludovic Croxford, PhD, from Edanz (https://jp.edanz.com/ac) for editing a draft of the manuscript.

## Author contributions

N.U. and H.M. conceived and designed the experiments. H.M. performed the evaluation micronucleation, MMCT efficiency, and chromosome tagging of Chr21, including the analyses following every experiment. K.Y. performed the chromosome tagging of ChrY. H.M. and M.E. performed the MMCT of ChrX and ChrY to hiPSCs. H.K. and K.K. performed long-term cell cultures and karyotyping. M. Osaki performed the histological analysis of the teratoma. T.S. produced the lentiviral vectors that expressed the ecotropic virus envelope. N.U., H.M., K.Y., S.H., K.T., M. Oshimura, and Y.K. wrote the manuscript.

## Declaration of interests

N.U., M. Oshimura, and Y.K. are inventors of patent applications based on the findings described in this paper. M. Oshimura is the CEO and shareholder of Trans Chromosomics Inc.

## Supplemental information

Data S1. Tables S1 and S7–S12

Document S2. Article plus supplemental information
