# Supplementary material for: Microcell-mediated chromosome transfer between non-identical human iPSCs
Source: Mol Ther Nucleic Acids. 2024 Nov 5;35(4):102382. doi: 10.1016/j.omtn.2024.102382 (PMC11616053; doi:10.1016/j.omtn.2024.102382)
Supplement: Document S1. Figures S1–S6, Tables S2–S6, S13, and S14 [file mmc1.pdf]

OMTN, Volume 35

Supplemental information

Microcell-mediated chromosome transfer between non-identical human iPSCs

Narumi Uno, Hitomaru Miyamoto, Kyotaro Yamazaki, Masaya Egawa, Hiroaki Kobayashi, Kanako Kazuki, Mitsuhiko Osaki, Teruhiko Suzuki, Shusei Hamamichi, Mitsuo Oshimura, Kazuma Tomizuka, and Yasuhiro Kazuki

Table S1. Summary of MMCT efficiency.

Table S2. Related to Figure 1E. Detailed results of the FISH analysis of HT1080 transferred with MAC6 from 201B7-MAC6 using Eco-MMCT

Exp #	Clone name	Major karyotype	Major karyotype ratio/analyzed cell (%)	HAC Maintenance ratio	Remarks
1	HT1080-MAC6-Eco#1-1	46+1	19/30 (63.3%)	0.97	
1	HT1080-MAC6-Eco#1-2	46+1	17/21 (80.9%)	0.95	
1	HT1080-MAC6-Eco#1-3	46+1	20/30 (66.6%)	0.80	
1	HT1080-MAC6-Eco#1-4	45+1	13/30 (43%)	0.93	-Y
2	HT1080-MAC6-Eco#2-1	46+1	24/30 (80.0%)	0.90	
2	HT1080-MAC6-Eco#2-2	46+1	28/30 (93.3%)	1.00	
2	HT1080-MAC6-Eco#2-3	46+1	12/30 (40.0%)	0.97	
2	HT1080-MAC6-Eco#2-4	45+1	22/30 (73.3%)	0.97	der(13;13)(q10;q10)
3	HT1080-MAC6-Eco#3-1	46+4	23/30 (76.6%)	1.00	
3	HT1080-MAC6-Eco#3-2	46+1	24/30 (80.0%)	1.00	
3	HT1080-MAC6-Eco#3-3	47+1	22/30 (73.3%)	1.00	20
3	HT1080-MAC6-Eco#3-4	45+1	19/30 (63.3%)	0.93	ins(7;MAC), -22?

Table S3. Related to Figure 4E. Detailed results of the FISH analysis of 585A1 transferred with Basal-HAC from 201B7-Basal-HAC using Eco-MMCT

Exp#	Clone name	Results [karyotyping/analyzed cell]
1	585A1-Basal-HAC#1-1	47,XY,+HAC[19/20]
1	585A1-Basal-HAC#2-2	47,XY,+HAC[19/20]
2	585A1-Basal-HAC#2-1	47,XY,+HAC[19/20]
2	585A1-Basal-HAC#2-2	48,XY,+HACx2[8/8]
2	585A1-Basal-HAC#2-3	47,XY,+HAC[18/19]
2	585A1-Basal-HAC#2-4	47,XY,+HAC[18/20]
3	585A1-Basal-HAC#3-1	50,XY,+HACx4[7/8]
3	585A1-Basal-HAC#3-2	47,XY,+HAC[16/20]
3	585A1-Basal-HAC#3-3	47,XY,+HAC[16/20]
3	585A1-Basal-HAC#3-4	47,XY,+HAC[19/20]

Table S4. Related to Figure 4F. Detailed results of the FISH analysis of 585A1 transferred with Chr21-tagged mCherry from 201B7 using Eco-MMCT

Exp#	Clone name	Results [karyotyping/analyzed cell]	Remarks
1	585A1-21Exp.1#01	48,XY,+21,+21 [7/8]	
2	585A1-21Exp.2#01	47,XY,+21 [8/8]	Teratoma, CGH array, Chromosome stability & Cell growth analysis
2	585A1-21Exp.2#02	46,XY [8/13], 48,XY,+21,+21[5/13]	
3	585A1-21Exp.3#01	48,XY,+21,+21 [8/8]	
3	585A1-21Exp.3#02	48,XY,+21,+21 [5/8], 47,XY,+21 [3/8]	
3	585A1-21Exp.3#03	47,XY,+21 [6/8]	
3	585A1-21Exp.3#04	47,XY,+21 [7/8]	Teratoma, Chromosome stability & Cell growth analysis
3	585A1-21Exp.3#05	47,XY,+21 [6/8]	
3	585A1-21Exp.3#06	47,XY,+21 [7/8]	
3	585A1-21Exp.3#07	47,XY,+21 [8/8]	

Table S5. Related to Figure 5E. Detailed results of the FISH analysis of 201B7 HPRT-deficient cell transferred with ChrX from 201B7 using Eco-MMCT

Exp#	Clone name	Results [karyotyping/analyzed cell]	Remarks
1	201B7 HPRT-KO-X Exp1-1	47,XX,+X [7/7]	Teratoma, CGH array, Chromosome stability & Cell growth analysis
2	201B7 HPRT-KO-X Exp2-1	48,XX,+X,+X [3/7], 46,XX [4/7]	Teratoma, Chromosome stability & Cell growth analysis

Table S6. Related to Figure 5F-5H. Detailed results of the FISH analysis of 201B7 transferred with ChrY-tagged GFPneo from SeV2-1-hY-GFPneo using Eco-MMCT

Exp#	Clone name	Results [karyotyping/analyzed cell]	Remarks
1	201B7-YGFPneoExp1-1	48,XX,+Y,+Y [11/20], 46,XX [8/20]	
1	201B7-YGFPneoExp1-2	48,XX,+Y,+Y [12/20], 46,XX [8/20]	
1	201B7-YGFPneoExp1-3	47,XX,+Y [17/20], 46,XX [3/20]	Teratoma, Chromosome stability & Cell growth analysis
1	201B7-YGFPneoExp1-5	48,XX,+Y,+Y [13/20], 46,XX [4/20]	
1	201B7-YGFPneoExp1-6	47,XX,+Y [15/20], 46,XX [5/20]	
1	201B7-YGFPneoExp1-7	48,XX,+Y,+Y [19/20], 46,XX [1/20]	
2	201B7-YGFPneoExp2-1	47,XX,+mar [12/20], 46,XX [8/20]	

3	201B7-YGFPneoExp3-1	47,XX,+Y [17/20], 46,XX [3/20]	Teratoma, CGH array, Chromosome stability & Cell growth analysis
3	201B7-YGFPneoExp3-2	48,XX,+Y,+Y [12/20], 46,XX [6/20]	
3	201B7-YGFPneoExp3-3	47,XX,+Y [19/20], 46,XX [1/20]	
3	201B7-YGFPneoExp3-4	47,XX,+mar [19/20], 46,XX [1/20]	

Table S7. Related to Figure 6A and 6B. Summary of the analysis of 585A1-21Exp.2#01 compared with 585A1

Table S8. Related to Figure 6C and 6D. Summary of the analysis of 201B7 HPRT-KO-X Exp1-1 compared with 201B7

Table S9. Related to Figure 6E and 6F. Summary of the analysis of 201B7-YGFPneoExp3-1 compared with 201B7

Table S10. Related to Figure 6. All data of the whole-genome CGH array analysis

Table S11. Sequence of EXON1 on the HPRT gene in 201B7 HPRT-KO

Table S12. Summary of the sequences targeted by CRISPR/Cas9 and plasmid vectors

Table S13. A list of primer sequences for plasmid construction and genomic PCR analysis

Name	Primer sequence (5' to 3')	Size (bp)	Description
21qTeloT9-mCherry-KI R-F1	AAGCTGCAATAAACAAGTTAAGC	670	Detection of Chr21 tagging
21qTeloT9-mCherry-KI R-R2	CCCTTGGTCAGATGGACACT	670	Detection of Chr21 tagging
ChrY HDR short-F	AAGTGTTGGCACAAAACAAGTAG	935	Detection of ChrY tagging
ChrY HDR short-R	GTGCTGGATATCTGCAGAATTCC	935	Detection of ChrY tagging
ChrY HDR long-F	AAGTGTTGGCACAAAACAAGTAG	10237	Detection of ChrY tagging
ChrY HDR long-R	GTTCTCTTTTTAACCCTTCCCCTTC	10237	Detection of ChrY tagging
mCat1fw1	accATGGGCTGCAAAAACCTGCTCG	1871	Constrution of pEF1-mCAT-1 expression vector
mCat1 rv2	TCATTTGCACTGGTCCAAGTTGCTGT	1871	Constrution of pEF1-mCAT-1 expression vector
mCat1 inf fw2	AGGTGTCGTGAGGAATTACCATGGGCTGCAAAAACCTG	1905	Constrution of pEF1-mCAT-1 expression vector
mCat1 inf rv2	CCTGAGGAGTGCGGCCTCATTTGCACTGGTCCAAGTTG	1905	Constrution of pEF1-mCAT-1 expression vector
DDX3Y F	GGGAAGAGAGAGTGCTTAAGGAAGAAG	615	Detection of the DDX3Y gene on ChrY
DDX3Y R	CACGAACCCACCAGAAGTGAAAC	615	Detection of the DDX3Y gene on ChrY
USP9Y F	ATAGATGGTGTGGAAAGACTTTTCTGGG	932	Detection of the USP9Y gene on ChrY
USP9Y R	CCTGCCCCAGCTTAGTAATTAACTCA	932	Detection of the USP9Y gene on ChrY
SRY F	ATGCAATCATATGCTTCTGCTATGTTAAGC	615	Detection of the SRY gene on ChrY

SRY F	CTACAGCTTTGTCCAGTGGCTG	615	Detection of the SRY gene on ChrY
HPRT sgRNA 01F	CCTCAGGCGAACCTCTCGGCTTTCC	428	Sanger sequencing for HPRT gene
HPRT sgRNA 01R	TGTCAACCAAAACGCCATTTCCACCTT	428	Sanger sequencing for HPRT gene
HPRT_01-NGS-Fw	TTCCTCCTCCTGAGCAACACTCTTTCCCTACACGACGCTCTTCCGATCTTTCCTCCTCCTGAGCAGTCA	N.A.	Next-generation sequencing
HPRT_01_NGS-Rv	GTGACTGGAGTTCAGACGTGTGCTCTTCCGATCTGCGTGACGTAAAGCCGAAC	N.A.	Next-generation sequencing
2ndF	AATGATACGGCGACCACCGAGATCTACAC-Index2-ACACTCTTTCCCTACACGACGC	N.A.	Next-generation sequencing
2ndR	CAAGCAGAAGACGGCATACGAGAT-Index1-GTGACTGGAGTTCAGACGTGTG	N.A.	Next-generation sequencing

Table S14. A list of primer sequences for STS marker analysis

Name	Sequence (5' to 3')	Size (bp)	Position (GRCh38/hg38)
DXS999 F	GCTAACAACCTAGACTTCAACC	268	chrX:18,817,245-18,816,978
DXS999 R	CAGTTTCACAATCTCTGCC	268	chrX:18,817,245-18,816,978
DXS1219 F	TTAATGTTTCANCCAGGTAAAT	230	chrX:32,063,082-32,063,311
DXS1219 R	GATCACTCCAAAGGATAGATTGT	230	chrX:32,063,082-32,063,311

DXS1105 F	TGTGAGGTTCAAGCCCC	212	chrX:108,300,874-108,300,663
DXS1105 R	GTTTCCGTGATTTACCATTTAGAC	212	chrX:108,300,874-108,300,663
DXS1227 F	AGAGGTCCGAGTCTTCCAC	174	chrX:141,714,259-141,714,432
DXS1227 R	ATAAGGGTTTACTCCCCCAA	174	chrX:141,714,259-141,714,432
DXS1073 F	GGCTGACTCCAGAGGC	233	chrX:154,600,879-154,600,647
DXS1073 R	CCGAGTTATTACAAAGAAGCAC	233	chrX:154,600,879-154,600,647
DXS7103 F	CACACACCCCTACCTGGA	128	chrX:9,609,418-9,609,291
DXS7103 R	CCCTAGAAGTTTTGCCCC	128	chrX:9,609,418-9,609,291

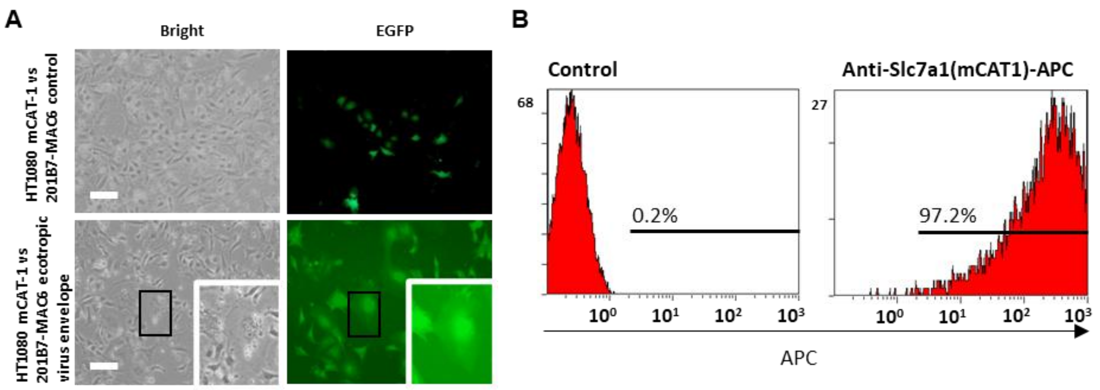

Figure S1. Application of the modified Eco-MMCT for hiPSC as CDC and CRC.

A, Representative images of the functional assay of the membrane fusion of chromosome donor and recipient cells via ecotropic virus envelope and mCAT-1 expressed by each cell type. Bright phase and GFP fluorescence images are shown. Co-culture with HT1080 expressing mCAT-1 as a receptor for the ecotropic virus envelope, and normal 201B7-MAC6 without ecotropic virus envelope (upper panels) and with ecotropic virus envelope (lower panels). The multinuclear syncytium was clearly observed by GFP. Scale bars represent 100 µm. B, FCM analysis of the expression of *Slc7a1* (mCAT-1) in each cell line used for chromosome recipients.

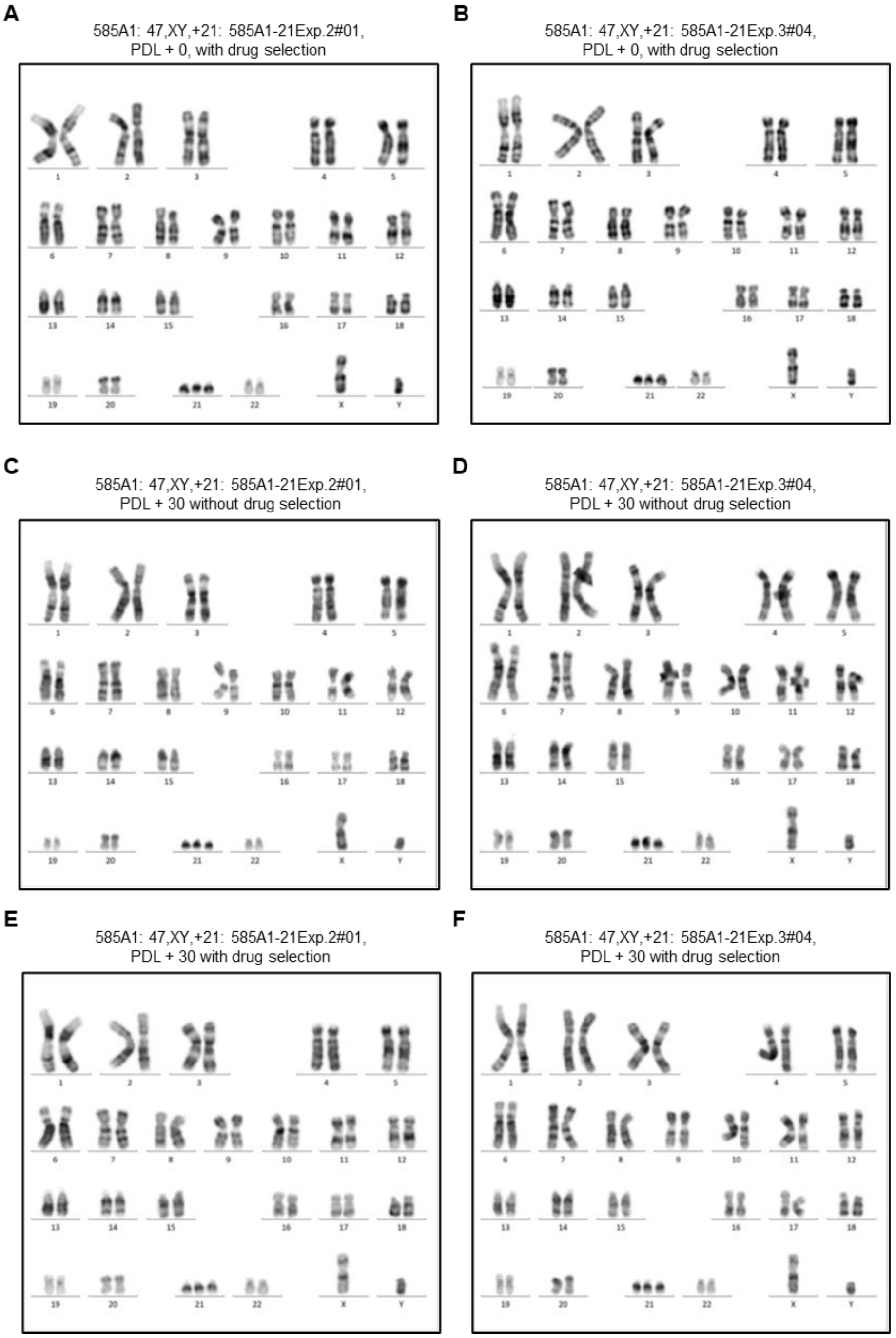

Figure S2. Related to Figure 7A. Q-banding karyotype analysis of 585A1 clones with transferred Chr21.

A and B, Karyotypes of 585A1-21Exp.2#01 and 585A1-21Exp.3#04 at PDL 0 with drug selection. C and D, Karyotypes of 585A1-21Exp.2#01 and 585A1-21Exp.3#04 at PDL 30 without drug selection. E and F, Karyotypes of 585A1-21Exp.2#01 and 585A1-21Exp.3#04 at PDL 30 with drug selection.

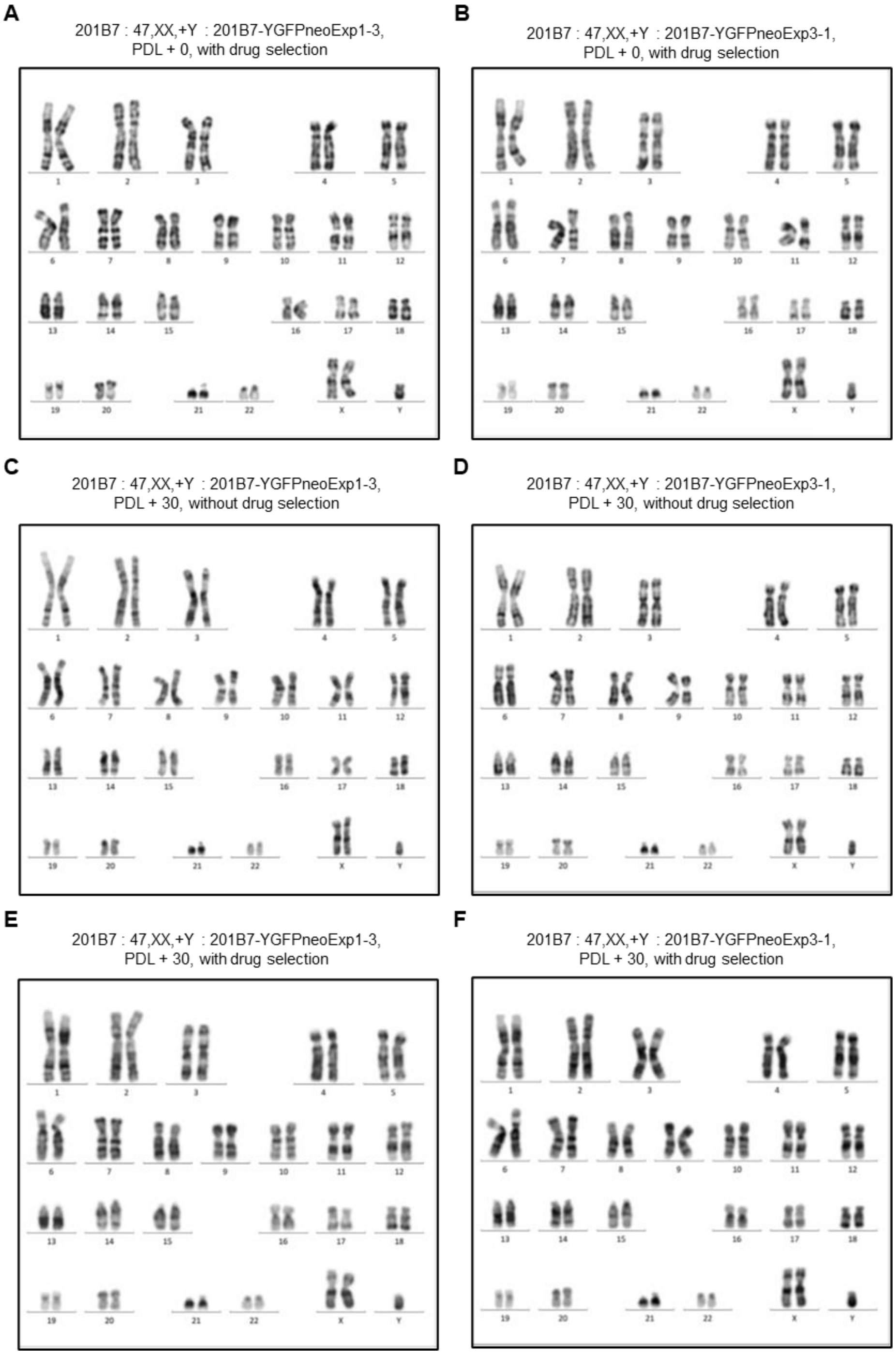

Figure S3. Related to Figure 7B. Q-banding karyotype analysis of 201B7 clones with transferred ChrY.

A and B, Karyotypes of 201B7-YGFPneoExp1-3 and 201B7-YGFPneoExp3-1 at PDL 0. C and D, Karyotypes of 201B7-YGFPneoExp1-3 and 201B7-YGFPneoExp3-1 at PDL 30 without drug.

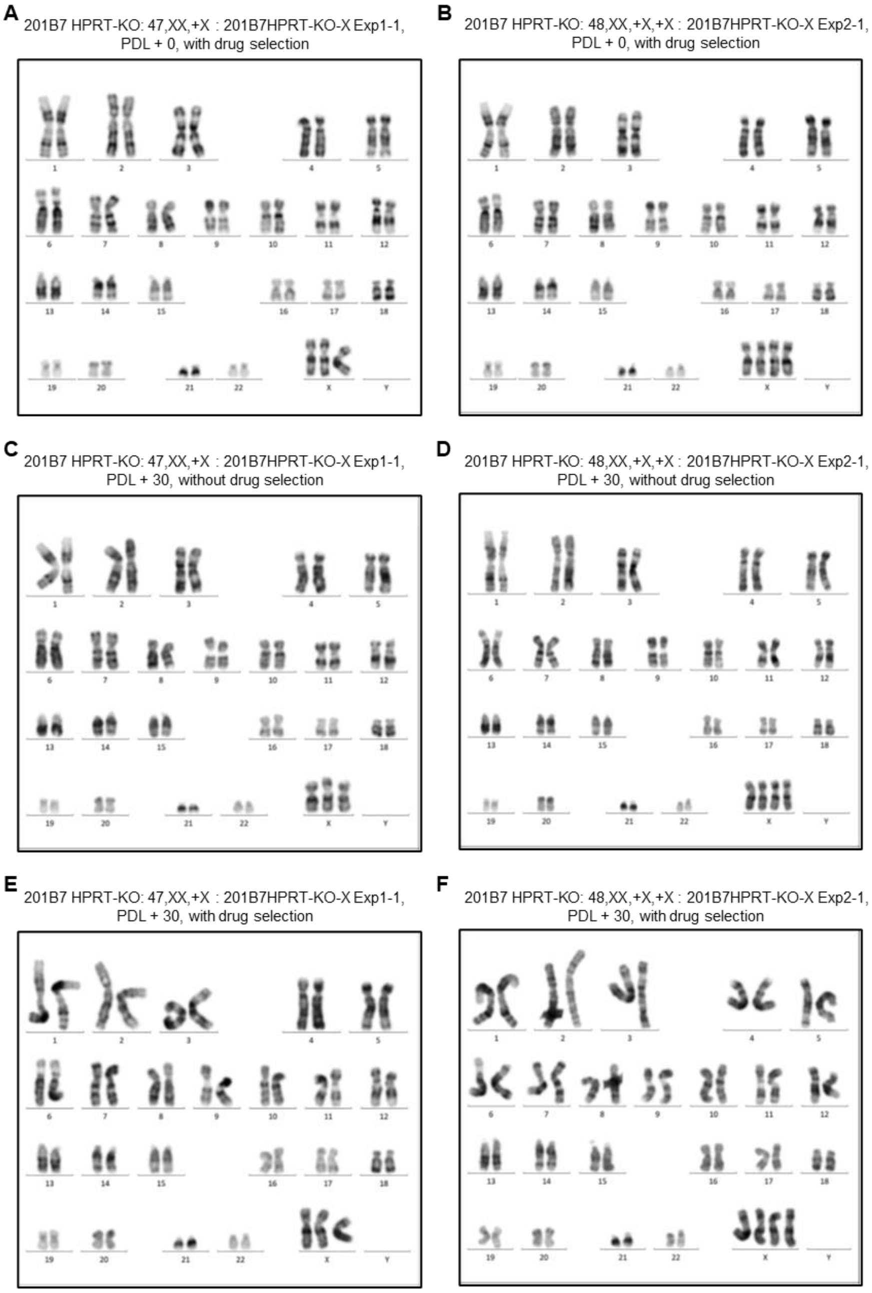

Figure S4. Related to Figure 7C. Q-banding karyotype analysis of 201B7 clones with transferred ChrX.

A and B, Karyotypes of 201B7 HPRT-KO-X Exp1-1 (47,XX,+X) and 201B7 HPRT-KO-X Exp2-1 (48,XX,+X,+X) at PDL 0. C and D, Karyotypes of 201B7 HPRT-KO-X Exp1-1 (47,XX,+X) and 201B7 HPRT-KO-X Exp2-1 (48,XX,+X,+X) at PDL 30 without drug selection. E and F, Karyotypes of 201B7 HPRT-KO-X Exp1-1 (47,XX,+X) and 201B7 HPRT-KO-X Exp2-1 (48,XX,+X,+X) at PDL 30 with drug selection.

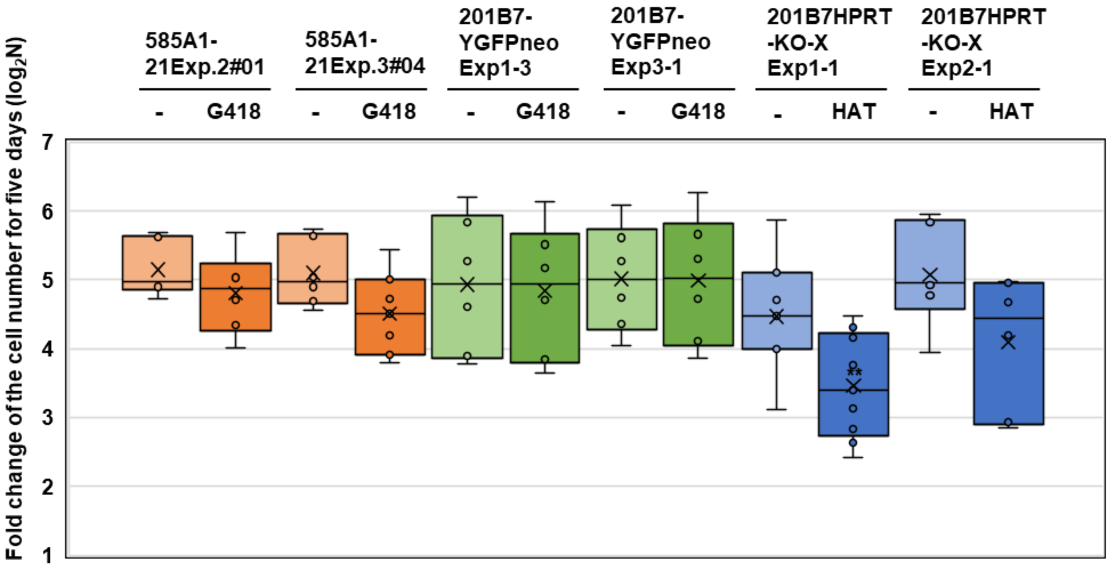

Figure S5. Related to Figure 7A-7D. Cell proliferation assay.

Comparison of the cell proliferation rates (fold-change of the cell number for five days, log2N) among clones with transferred Chr21, ChrY, and ChrX, under conditions with and without drug selection The number (*n*) for each condition from left to right is as follows: *n* = 6, 6, 6, 7, 6, 6, 6, 6, 7, 9, 6, and 6 passage.

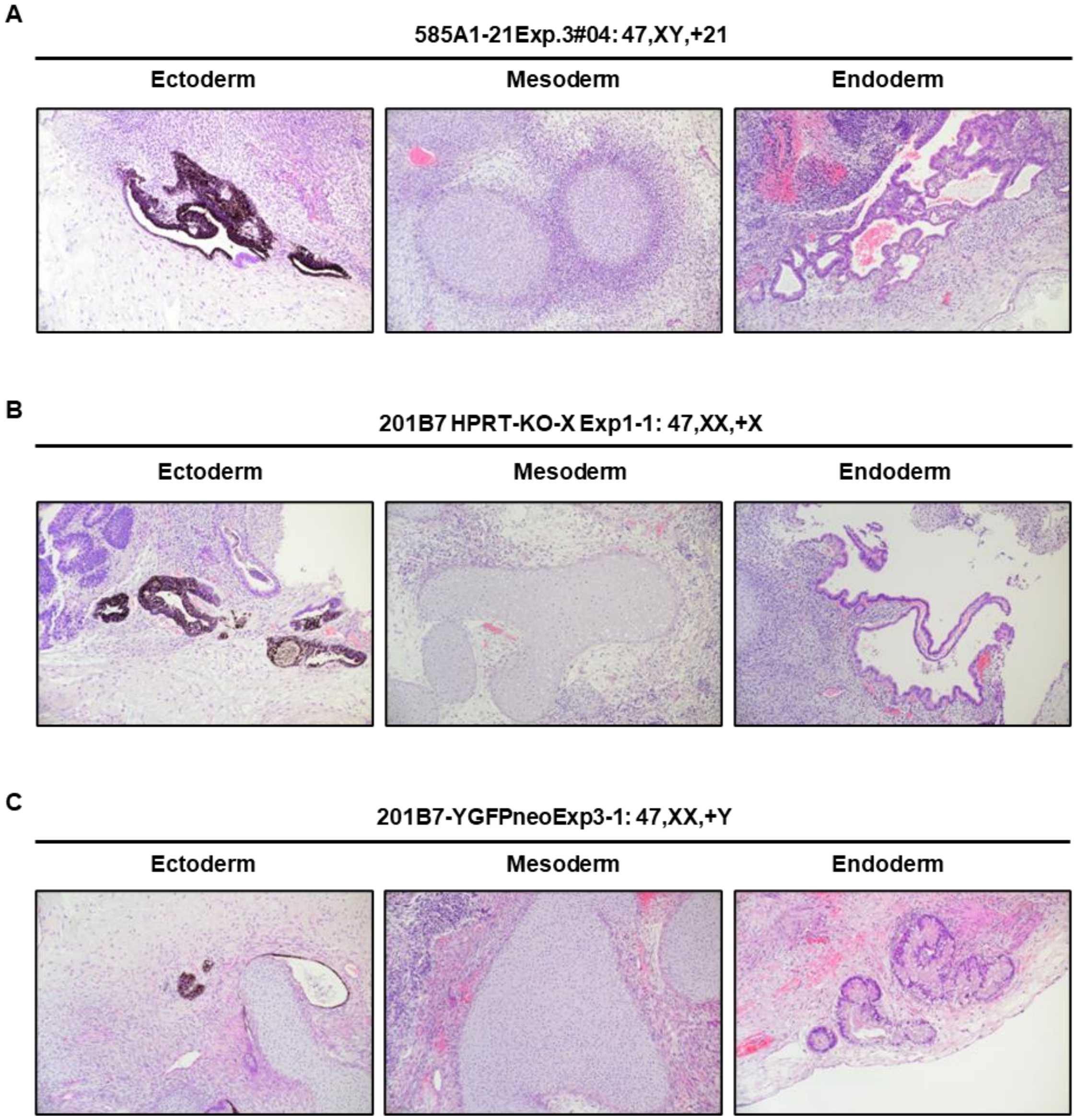

Figure S6. Teratoma formation assay and histology to evaluate trilineage differentiation.

A, Representative images of the teratoma formation assay showing the trilineage differentiation of A, 585A1-21Exp.3#04, B, 201B7 HPRT-KO-X Exp1-1, and C, 201B7-YGFPneo Exp3-1.
